# Kinetics of enzyme-catalysed desymmetrisation of prochiral substrates: product enantiomeric excess is not always constant

**DOI:** 10.3762/bjoc.17.73

**Published:** 2021-04-21

**Authors:** Peter J Halling

**Affiliations:** 1 WestCHEM, Dept Pure & Applied Chemistry, University of Strathclyde, Glasgow G1 1XL, Scotland, UK

**Keywords:** enantiomeric excess, enzyme, kinetic mechanisms, kinetic parameters, prochiral

## Abstract

The kinetics of enzymatic desymmetrisation were analysed for the most common kinetic mechanisms: ternary complex ordered (prochiral ketone reduction); ping-pong second (ketone amination, diol esterification, desymmetrisation in the second half reaction); ping-pong first (diol ester hydrolysis) and ping-pong both (prochiral diacids). For plausible values of enzyme kinetic parameters, the product enantiomeric excess (ee) can decline substantially as the reaction proceeds to high conversion. For example, an ee of 0.95 at the start of the reaction can decline to less than 0.5 at 95% of equilibrium conversion, but for different enzyme properties it will remain almost unchanged. For most mechanisms a single function of multiple enzyme rate constants (which can be termed ee decline parameter, *eeDP*) accounts for the major effect on the tendency for the ee to decline. For some mechanisms, the concentrations or ratios of the starting materials have an important influence on the fall in ee. For the application of enzymatic desymmetrisation it is important to study if and how the product ee declines at high conversion.

## Introduction

There is great interest in using enzymatic catalysis in the synthesis of homochiral molecules. An early approach was to use enantioselective enzymes in the resolution of a racemate. As such resolution reactions proceed, there are progressive changes in the enantiomeric excesses (ee), that of the product falling while that of the residual starting material increases. In a classic study of the kinetics of resolution, Chen and Sih [1] showed how these changes reflected an unchanging characteristic of the enzyme, the enantioselectivity *E*, equal to the ratio of *k*_cat_/*K*_m_ values for the two enantiomers. The Chen and Sih equations are widely used to analyse the progress of resolution reactions. Chen and Sih also described the influence of the equilibrium constant where the enzymatic reaction was not completely irreversible [2].

A limitation of the enzymatic resolution is that the maximum yield of the desired enantiomer is the 50% contained in the starting racemate. This is one reason for the current greater interest in enzymatic desymmetrisation reactions, in which a prochiral substrate is used [3–7]. The reaction generates a new chiral centre, and the enzyme shows enantiospecifity in making predominantly one enantiomer as the product. In this case the yields of the favoured enantiomer can approach 100%. Some popular reactions are the reduction of prochiral ketones to chiral secondary alcohols, transamination of prochiral ketones to chiral amines, hydrolysis of symmetrical diesters to a chiral monoester, and esterification of prochiral diacids or diols.

In desymmetrisation reactions, the enzyme initially produces the two product enantiomers in an unchanging ratio (with one usually strongly favoured). Hence it is usual to quote the enantiomeric excess (ee) of the product as if it were a characteristic property of the enzyme under the given conditions, remaining constant up to high conversions. A search for all publications over the last year or so dealing with the enzymatic desymmetrisation of prochiral compounds showed that all quote a value of ee to characterise the behaviour for any enzyme and reaction conditions [8–31]. Sometimes this comes with a conversion or yield at which it was obtained. However, I have heard anecdotally from people performing such reactions that the ee can appear to decline as the reaction proceeds. (I have been unable to find any published report of this effect).

Perhaps because of the view that the kinetics of such reactions are straightforward, they have received little study. Smith et al. [32] and more recently, Yamane [33] have studied the kinetics of example reactions, but without progress data for the ee. Kroutil et al. [34] derived a model for consecutive reactions, but the desymmetrisation reaction was assumed to produce enantiomers in a fixed ratio throughout. The current paper presents a study of the kinetics of enzymatic desymmetrisation reactions that explicitly examines the progress of the ee. It shows that for plausible enzyme parameters, the product ee can decline at high conversion, often quite substantially. Understanding this effect, and features of the enzyme and reaction conditions that can minimise it, is important for the optimal design of enzymatic desymmetrisation reactions.

## Results and Discussion

**Scheme 1:**
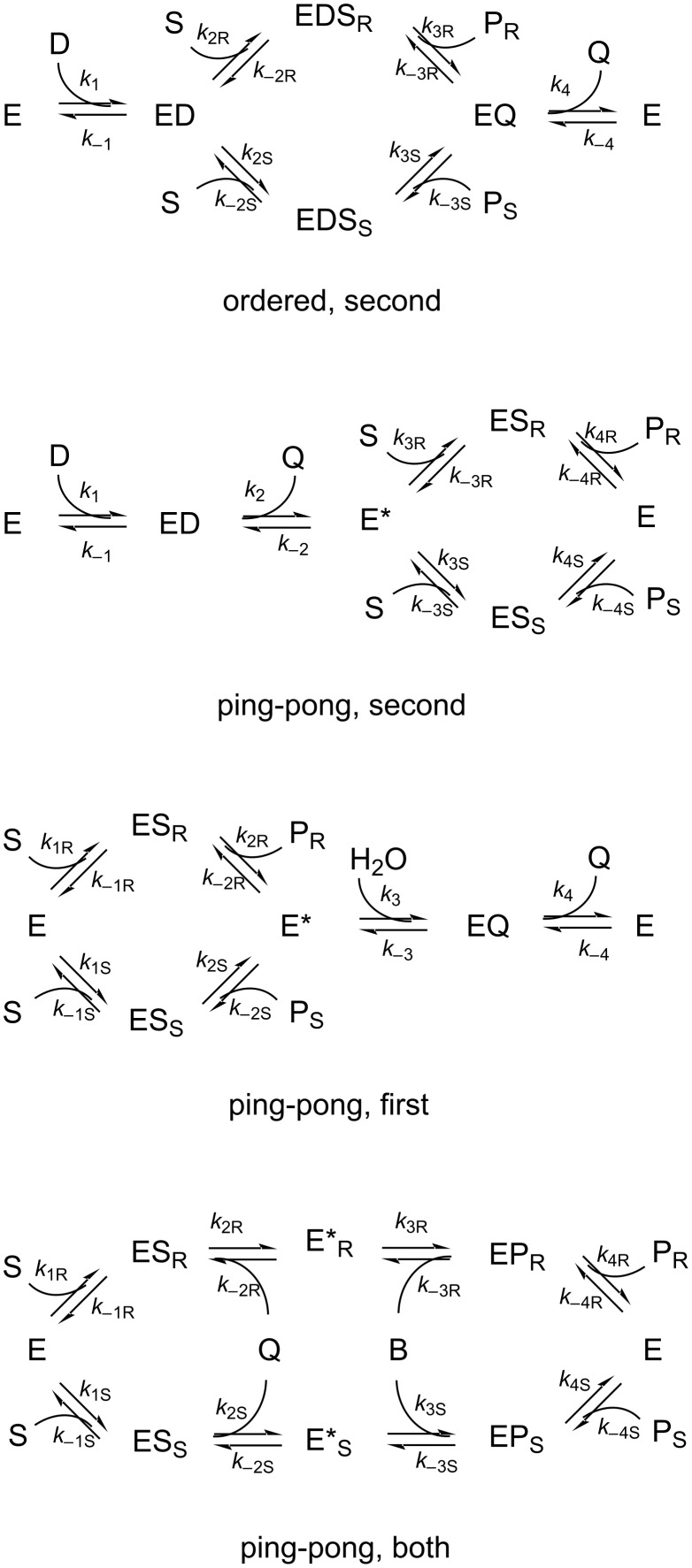
Kinetic mechanisms. In each case E represents the free enzyme, other species starting E are other enzyme forms, S is the prochiral substrate, D, B or H_2_O is the second substrate, Q is the side-product, and P_R_ and P_S_ are the two enantiomeric chiral products. Elementary kinetic rate constants are shown next to each reaction arrow.

Scheme 1 shows the kinetic mechanisms that cover most applications of enzymatic desymmetrisation. These show that the mechanisms necessarily include a pathway of elementary reactions catalysed by the enzyme that brings about racemisation of the product. This will always be possible unless either the reaction is completely irreversible, or the reactions leading to the less favoured product are completely absent. In most of the applications the overall reaction is significantly reversible, with an equilibrium constant that is not enormously larger than 1. And in many cases there is a noticeable formation of the less favoured enantiomer, with product ee values of 0.98 or less. Even an ee of >0.99, as often reported, may not be enough to make formation of the less favoured enantiomer always negligible. It should be clear that the formation of a product with a fixed ee is an initial rate phenomenon, which may not persist to high conversion. Eventually the ee must fall towards the ultimate equilibrium value of 0, although the timescale for this depends on details of the enzyme kinetics.

**Figure 1:**
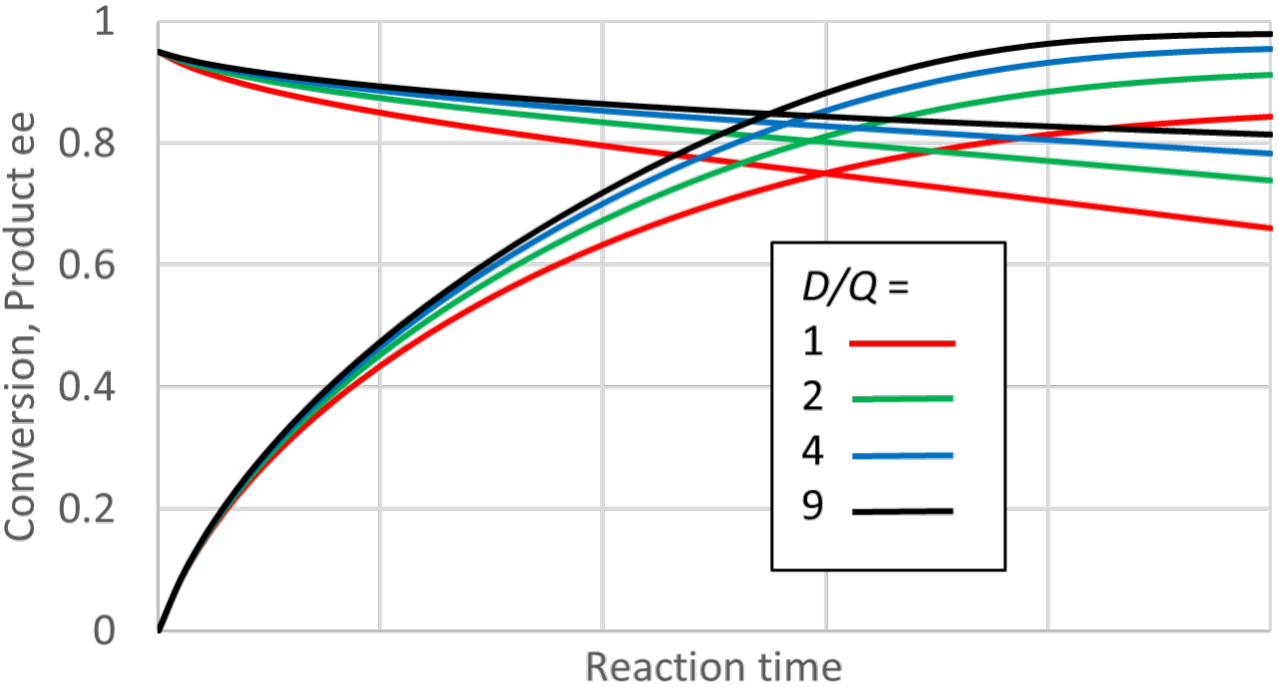
Reaction progress for “ordered, second” kinetics and the effect of *D*/*Q* (e.g., NADH/NAD^+^) ratio. *S*_0_ = 0.2 M, total D + Q = 0.01 M, *K*_eq_ = 5, *E* = 39, *K*_MD_ = 0.001 M, *K*_MS_ = 0.001 M, *k*_4_ = 1000 s^−1^, *SCRf*/*b* = 1, *k*_3R_/*k*_4_ = 1, *k*_3R_/*(E · k*_3S_*)* = 1, *k*_−4_/*k*_1_ = 1, *k*_−2R_/*k*_−1_ = 1, *E · k*_−2S_/*k*_−1_ = 1. For the calculation the enzyme concentration was adjusted such that all initial rates were the same. Hence the reaction time is essentially dimensionless, and no values are given on the axis. The *K*_eq_ value here is found for reduction of simple ketones by NADH at pH around 7.5 [35].

To understand the range of possible behaviour, progress curves for the conversion and the ee were calculated for a wide variety of possible enzyme kinetic parameters. The calculation used integration of the fundamental differential equations that describe the relevant kinetic mechanism. For some plausible kinetic parameters, the results show a substantial decline in the product ee as the reactions are run to high conversion. Figure 1 below shows some examples. It is clear that in such cases a single value of the product ee does not characterise the enzyme and reaction behaviour. An ee value observed under some particular circumstances may be a poor guide to performance under others. In particular, a good ee found early in the reaction may be a poor guide to a preparative reaction where a high conversion is desirable.

The details of behaviour depend on the kinetic mechanism followed by the enzyme and the reaction of interest. Most of the reported examples will follow one of the 4 kinetic mechanisms shown in Scheme 1. The behaviour is somewhat different in each case, so these kinetic mechanisms need to be considered separately. For reference in this paper, the kinetic mechanisms are labelled using two words, referring to the overall type and the location of the enantiospecific step(s).

- “Ordered, second”. This is the ternary complex ordered mechanism followed by most dehydrogenases and keto-reductases. The reductant (usually NADH or NADPH) has to bind first to the enzyme, followed by the prochiral ketone in the second step. The chiral products are then released before the oxidised co-product.
- “Ping-pong, second”. Followed by most transaminases and lipase or esterase-catalysed acylation of prochiral diols. The enzyme reacts with an amino or acyl donor, releasing a first co-product, to give an aminated or acylated enzyme intermediate. This then reacts with the prochiral ketone or diol to generate the chiral products.
- “Ping-pong, first”. Followed in most lipase or esterase hydrolyses of prochiral diol esters. The enzyme reacts enantiospecifically with the ester to release a chiral product, leaving the acyl group attached to the active site. In a second stage the achiral acyl group undergoes hydrolysis by water. In the desymmetrisation of diols (and diacids, below) kinetic amplification can sometimes be exploited to raise the product ee [7]. This involves the selective further reaction of the unwanted product enantiomer. Including kinetic amplification in the model would involve doubling the number of enzyme rate constants, which was considered excessive complication at this stage.
- “Ping-pong, both”. Followed in most lipase or esterase reactions of prochiral diacids: either hydrolysis of their esters, or esterification of the free acids. The enzyme reacts enantiospecifically with the ester or acid. But now the acyl enzyme can be in either stereoisomeric form, so the kinetics are different for all subsequent steps.

It might be hoped that behaviour in each of these cases could be predicted intuitively based on the extensive published analysis of the kinetics of the ternary complex ordered and ping-pong mechanisms. However, I was unable to do so. The effects reported were mainly identified by empirical observation of the results of simulating enzyme reaction progress, varying input parameters over likely ranges. Some effects could be rationalised after they had been identified.

The next section explains the selection of parameters that describe enzyme properties important in determining the time course of the product ee. Readers who are mainly interested in seeing the possible behaviour can skip this section, perhaps coming back later to see the derivation of enzyme parameters.

### Enzyme parameters affecting behaviour

The overall kinetic behaviour is governed by at least 12 elementary rate constants that appear in the appropriate kinetic mechanism. The reaction progress was determined by integrating the differential equations incorporating these rate constants. The value of each of these rate constants depends on the enzyme and reaction conditions chosen for use.

However, it is more informative to consider the behaviour as a function of groups that each combine several elementary rate constants, as normal in enzyme kinetics. Firstly, there are constraints that mean the enzyme cannot vary all these rate constants independently. A Haldane relationship makes a combination of rate constants equal to the equilibrium constant of the overall reaction, which the enzyme is of course unable to change. A different combination of rate constants must equal the equilibrium constant for the isomerisation of one product enantiomer into the other, which is necessarily 1 (in an achiral medium).

It would thus be possible to treat all but 2 of the rate constants as independent input parameters. However, this is not the most sensible approach. Instead, combinations of rate constants can be related to values more likely to be known by users of relevant enzymes. And other combinations prove to have a dominant influence on the type of behaviour observed, so that this can be largely captured using fewer input parameters.

Almost always the ratio of product enantiomers formed in the initial stages of a reaction will be known from measurements, so this is a sensible input parameter, equal to a combination of elementary rate constants. Often estimates will be available for the *K*_M_ values of the enzyme under initial rate conditions in the forward direction. These *K*_M_ values can also be used as input parameters, again equal to a combination of rate constants. Finally, multiplying all elementary rate constants by the same factor will not change the shape of the progress of product formation and enantiomeric excess, just the timescale over which this occurs. Hence one elementary rate constant may be set to an arbitrary value, with others effectively expressed as a multiplier times this value. This can be seen as setting the timescale for the reaction.

This leaves at least 6 more independent parameters that determine the enzyme behaviour. The grouping of elementary rate constants to define these parameters is a matter for judgment. The ones selected in this study were based in part on a mathematical analysis of the rate equations, using the usual quasi-steady state approximation. This showed that certain groupings were common in the derived equations for the progress of product formation. Parameter choices were also influenced by empirical observation of effects on the decline in product ee as the reaction progressed. It proved possible to choose sets of parameters such that most of the influence came from the values of just a few parameters, with others having little effect. Hence most of the behaviour could be understood in terms of just a few parameters, giving a simpler overall picture. A further criterion was a preference for dimensionless parameters, like the ratio of two elementary rate constants (both first order or both second order). Dimensionless parameters make it easier to represent all possible behaviour of diverse enzymes. Finally, many parameters could be defined in such a way that they were expected to be usually of the order of magnitude of 1, so assigning values for kinetic simulations was easier.

One type of grouping of rate constants was found to come up regularly in the analytical solution of equations resulting from the quasi-steady state approximation. These had the form of a *k*_cat_/*K*_M_ ratio (a specificity constant), but just for a part of the overall reaction. For example, in a ping-pong reaction, if we think of the first two steps independently, the *K*_M_ would be (*k_−_*_1_ + *k*_2_)/*k*_1_, and the *k*_cat_ would be *k*_2_, so the specificity constant equivalent is *k*_1 _*· k*_2_/(*k*_−1_ + *k*_2_). This is the sort of grouping that came out from the equations. It is important to note, however, that these are not equal to the true *k*_cat_ and *K*_M_ of the enzyme that might be measured by study of the overall reaction. Hence they are referred to as pseudo-specificity constants. They are labelled by “*SC*”, usually followed by *R* or *S* (for the two enantiomers), then *f* or *b* to indicate the forward or backwards progress through the relevant two steps. SC values were normally used for the two steps that involve enantioselectivity (2 and 3 for the ordered, second mechanism, 3 and 4 for ping-pong, second, 1 and 2 for ping-pong, first). For the ping-pong, both mechanism, there are two sets of SC values, *SCRf* etc. for steps 1 and 2, and *SCR2f* etc. for steps 3 and 4.

These pseudo-specificity constants have units of M^−1^ s^−1^, so were not used directly as input parameters to simulations. However, their ratios were used, sometimes in combination with other parameters. So for example the ratio *SCRf*/*SCSf* was found to give (in most cases) the ratio of initial rates of formation of the two enantiomer products. Hence it can be named as an *E* value for the enzyme, by analogy with the enantioselectivity in resolution reactions. Similarly, the ratio *SCRf*/*SCRb* was found to have an important influence in all cases – it is given the symbol *SCRf*/*b*. For the ping-pong, both mechanism, the ratio *SCR2f*/*SCS2f* is also relevant, and was given the symbol *E2*.

### Behaviour for “ordered, second” kinetics

This is the kinetic model expected for most dehydrogenases and ketoreductases carrying out reduction of prochiral ketones. In such reactions it is usual to recycle the co-substrate, usually NADH or NADPH. To give an idea of the behaviour under co-substrate (cofactor) recycling, the reaction progress was simulated with concentrations of D (NAD(P)H) and Q (NAD(P)^+^) constant throughout the reaction. This is a fair approximation for what happens with efficient recycling. Figure 1 shows the simulated progress of a reaction for a set of plausible conditions and enzyme properties. In all cases the product ee falls noticeably from the initial value of 0.95 as the reaction progresses. This decline is substantially faster for lower values of the ratio of D to Q. As this ratio decreases, the progress of conversion also slows more, as expected, as the reaction approaches a lower equilibrium conversion. The enzyme parameter values chosen for Figure 1 are ones for which the ee does decline significantly; the range of possible behaviour is presented below. The input parameter values are defined for the case where the *R* enantiomer is favoured. But the behaviour is actually completely symmetric, and simply exchanging all *R* for *S* and vice versa covers cases where *S* is favoured.

To aid comparison of reactions under different conditions and enzyme parameters, we can examine the product ee at a conversion that is 95% of that expected at equilibrium (for a fully enantiospecific reaction). This is a typical conversion that might be selected for a preparative reaction. For the reactions in Figure 1, these ee values are 0.725, 0.782, 0.816, and 0.836 as D/Q is increased from 1 to 9. All of these ee values are of course substantially lower than 0.950 found at the beginning of the reaction. Under cofactor recycling, the ratio D/Q can be controlled by the excess of ultimate reductant chosen, and also by its identity. Changing the equilibrium constant of the reaction will also affect the equilibrium conversion, but has a much smaller effect on the product ee. For D/Q = 2, a doubling the *K*_eq_ from 5 to 10 only increases the ee at 95% of equilibrium conversion from 0.782 to 0.793. Changing the total concentration of D + Q had a negligible effect, provided the ratio remained the same.

**Figure 2:**
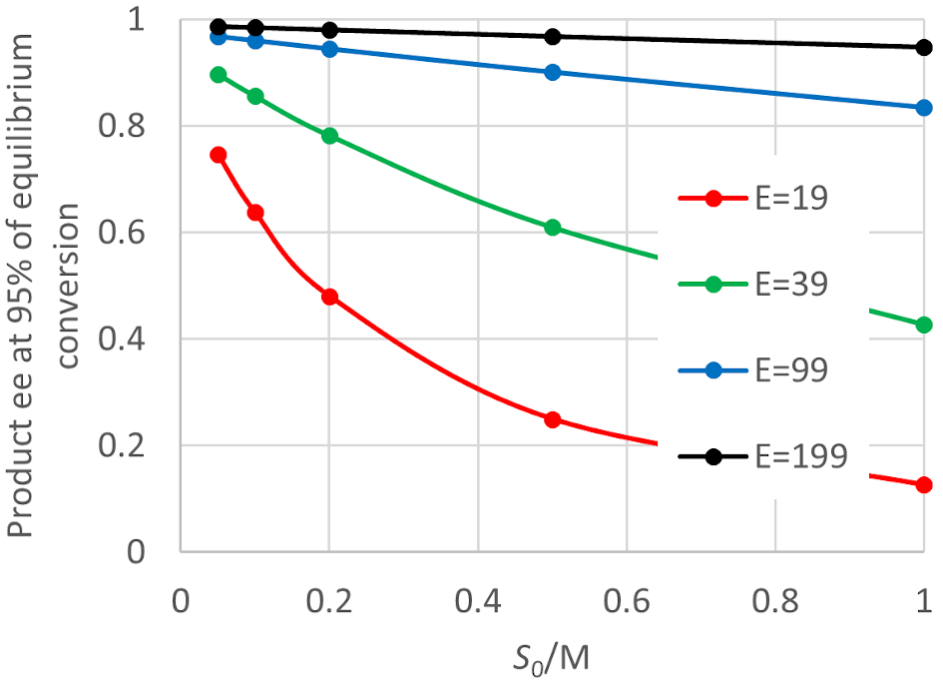
Effect of the initial starting material concentration and enzyme *E* value for “ordered, second” kinetics. D = 0.00333 M, Q = 0.00167 M, *K*_eq_ = 5, *E* = 39, *K*_MD_ = 0.001 M, *K*_MS_ = 0.001 M, *k*_4_ = 1000 s^−1^, *SCRf*/*b* = 1, *k*_3R_/*k*_4_ = 1, *k*_3R_/*(E · k*_3S_*)* = 1, *k*_−4_/*k*_1_ = 1, *k*_−2R_/*k*_−1_ = 1, *E · k*_−2S_/*k*_−1_ = 1.

The example of Figure 1 was for a case where the initial product ee was 0.95 (*E* = 39), because it is easier to see changes in ee on the graph. Figure 2 shows what happens for other *E* values, and also the effect of the initial concentration of the prochiral starting material, which was found to have an important effect. As can be seen there are noticeable falls in the product ee by the time the reaction approaches equilibrium, for all *E* values. At a starting material concentration of 1 M, the product ee falls from the values early in the reaction of 0.99 (*E* = 199) and 0.98 (*E* = 99) to 0.948 and 0.834, respectively. For fixed enzyme properties, the product ee near equilibrium also falls noticeably with an increase in the prochiral starting material concentration. It would be quite common for a reaction found to be useful at low concentration to be repeated at a higher concentration for preparative purposes. As this graph shows there might be an unwelcome and unexpected fall in the product ee obtained. To study the effects of other enzyme parameters, simulations were routinely run for an *E* value of 39 (initial product ee 0.95), which makes changes clearer to display.

A number of other enzyme parameters were found to have negligible effects on the product ee at 95% equilibrium conversion: *K*_MD_, *k*_−2R_/*k*_−1_, *E · k*_−2S_/*k*_−1_. As expected, *k*_4_ has absolutely no effect, simply making all changes happen faster or slower – so changes in *k*_4_ are compensated by changes in simulated enzyme concentration, in order to keep the normalised fixed initial rate.

**Figure 3:**
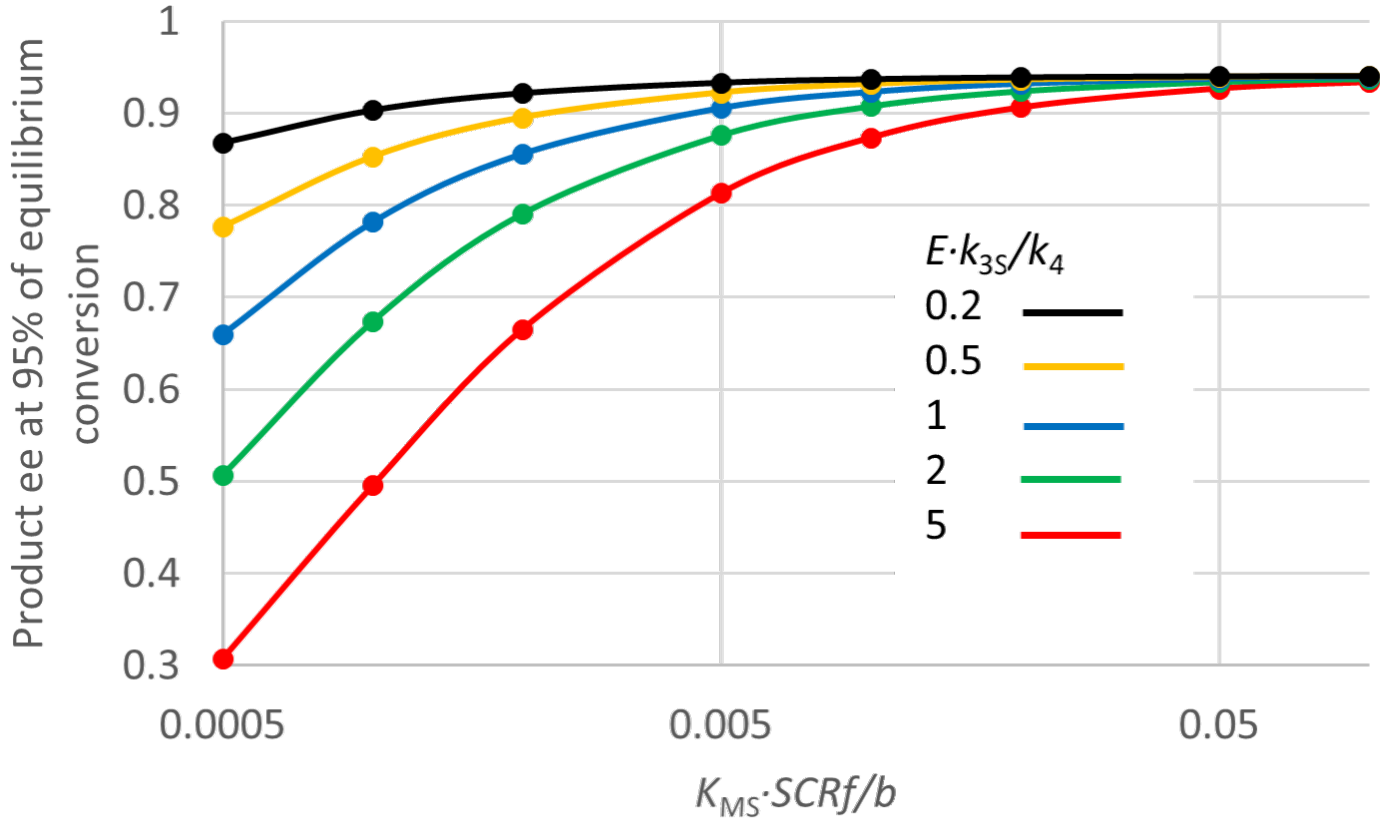
Effects of key enzyme parameters on the fall in product ee during reaction for “ordered, second” kinetics. *S*_0_ = 0.2 M, D = 0.00333 M, Q = 0.00167 M, *K*_eq_ = 5, *E* = 39, *K*_MD_ = 0.001 M, *K*_MS_ = 0.001 M, *k*_4_ = 1000 s^−1^, *k*_3R_/*k*_4_ = 1, *k*_−4_/*k*_1_ = 1, *k*_−2R_/*k*_−1_ = 1, *E · k*_−2S_/*k*_−1_ = 1. For this plot the groups shown were varied by changing *SCRf*/*b* and *k*_3R_/(*E · k*_3S_). However, the values of ee at 95% of equilibrium conversion were almost identical if the values of the same groups were varied by changing *K*_MS_ and *k*_3R_/*k*_4_.

The effects of the remaining enzyme parameters could be largely summarised by two groupings, the product *K*_MS _*· SCRf*/*b* and the ratio *E · k*_3S_/*k*_4_ (actually the ratio of input parameters *k*_3R_/*k*_4_ and *k*_3R_/(*E · k*_3S_)). This behaviour is shown in Figure 3. As can be seen, if *K*_MS _*· SCRf*/*b* is sufficiently large, and *E · k*_3S_/*k*_4_ is sufficiently small, the product ee will remain high throughout the reaction, remaining around 0.94 even at 95% of the equilibrium conversion, only slightly less than its initial value of 0.95. However, if the enzyme does not have these properties, the product ee can drop substantially as the reaction progresses, even to values that are of little preparative value. In fact, most of the effect of these two parameters is captured by their ratio, so the group *E · k*_3S_/*k*_4_ divided by *K*_MS _*· SCRf*/*b* acts as a single parameter that describes the tendency for the ee to decline with a particular enzyme, which can be referred to as “ee decline parameter (*eeDP*)”. If the value of *eeDP* for a particular enzyme is small, the decline in ee at high conversion will be small and perhaps negligible. However, for an *eeDP* greater than 100 M^−1^, and particularly greater than 1000 M^−1^, the product ee at preparative conversion can be substantially lower than expected from the *E* value. Hence it would be valuable to estimate the *eeDP* for candidate enzymes. Measurements of the product ee at 2 or 3 different conversions may be sufficient to give a rough estimate, which would be valuable for planning a synthesis. When selecting the best enzyme, the *eeDP* may be as important to know as the *E* value. Note that *eeDP* values greater than 100 M^−1^ are probably quite common, due to a *K*_MS_ of less than about 0.01 M. It would also be possible to give the *eeDP* units based on mM, so that the limiting values become 0.1 and 1 mM^−1^. Clearly the *eeDP* is likely to be large where *K*_MS_ is small. Intuitively a low *K*_MS_ for the prochiral starting material might be thought a good thing, and it may well be in terms of the reaction rate. But this analysis shows that it is undesirable in terms of maintaining a good product ee through the reaction progress. One other enzyme parameter, *k*_−4_/*k*_1_, had a smaller but noticeable effect on the decline in product ee, with higher values making this worse (greater).

When the simulation parameters incorporated in the *eeDP* are expanded in terms of elementary rate constants, some cancellations occur:


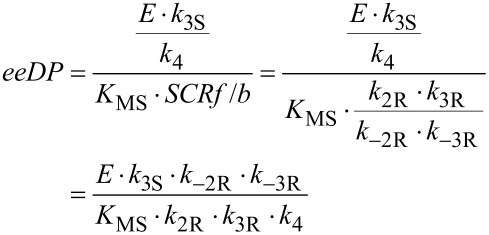


It is possible also to express *E* and *K*_MS_ in terms of elementary rate constants and substitute in this expression, but the result does not offer any clear interpretation (see Supporting Information File 1). It makes intuitive sense that higher values of backwards rate constants for the R enantiomer will increase the tendency for the ee to decline as the reaction approaches equilibrium. The ratio *k*_−2R _*· k*_−3R_/(*k*_2R _*· k*_3R_) will tend to be greater for smaller values of *K*_eq_, but the relationship is not automatic, as the full expression for *K*_eq_ also includes *k*_−1 _*· k*_−4_/(*k*_1 _*· k*_4_). A change in *K*_eq_ necessarily means a change in some elementary rate constants, but the enzyme properties determine the details of these changes, subject to maintaining the overall thermodynamic relationship. With the parameter set used in these simulations, an increase in *K*_eq_ alone mainly caused reductions in *k*_−1_ and *k*_−2R_, partly offset by an increase in *k*_−3R_.

The examples of Figure 2 and Figure 3 above were for a case where the *eeDP* was 1000 M^−1^, so where the decline in ee was relatively strong. This was chosen in order to make the effects clearly visible. It is possible that many enzymes used in practice have values of the ratio such that falling ee is not a major issue. But the values chosen here are not exceptional, and many enzymes used in practice are likely to face some problem with a decline in ee at high conversion.

It is unlikely that people working with these enzymes will start with an idea of the magnitude of *eeDP* or all the parameters incorporated in it. However, the *eeDP* might be estimated by accurate data for the product ee at different stages of the reaction and for different starting material concentrations. That would allow predictions for what product ee might be found under different conditions. It should be clear from the behaviour shown here that quoting a single value of product ee can be very misleading about how an enzyme will perform under different conditions.

### Behaviour for “ping-pong, second” kinetics

**Figure 4:**
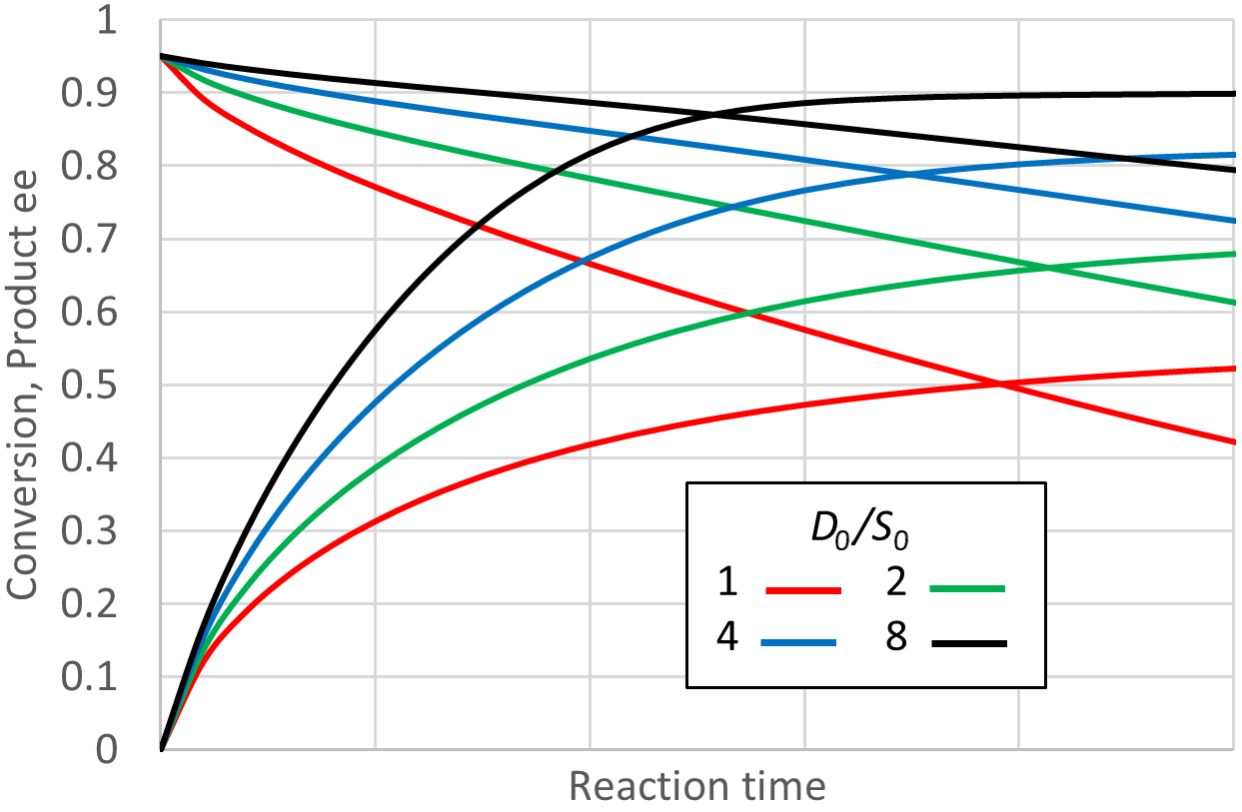
Reaction progress for “ping-pong, second” kinetics, and the effect of the ratio of donor to prochiral substrate. *S*_0_ = 0.2 M, *K*_eq_ = 1, *E* = 39, *K*_MD_ = 0.2 M, *K*_MS_ = 0.01 M, *k*_2_ = 1000 s^−1^, *SCRf*/*b* = 1, *k*_2_/*k*_4R_ = 1, *E · k*_4S_/*k*_4R_ = 1, *k*_−1_/*k*_2_ = 1, *k*_3R_/*k*_−4R_ = 1, *k*_3S_/*k*_−4S_ = 1. Again the enzyme concentration was adjusted such that all initial rates were the same. The *K*_eq_ will be close to 1 for many transaminase or transesterification reactions, but may be substantially higher, if activated donors are used.

This kinetic mechanism should apply for most lipase or esterase-catalysed acylations of a prochiral diol. It will also apply to most transaminases used with a prochiral ketone as amino group recipient. Figure 4 shows some progress curves for this mechanism with plausible values of enzyme parameters (admittedly chosen such that the ee does decline, see below). As can be seen, for these parameters the product ee falls quite rapidly from its initial 0.95 as the reaction proceeds. The decline is less, if there is a higher ratio of D (donor of, e.g., amino or acyl groups) to S, the prochiral starting material (ketone or diol). The higher ratio also increases the equilibrium conversion, of course, and shortens the time to reach it. Hence the product ee values at 95% of the equilibrium conversion are 0.687, 0.768, 0.840, and 0.890 for *D*_0_/*S*_0_ of 1, 2, 4, and 8, respectively. These values were unaffected by the actual concentration *S*_0_, provided *D*_0_/*S*_0_ was kept the same.

**Figure 5:**
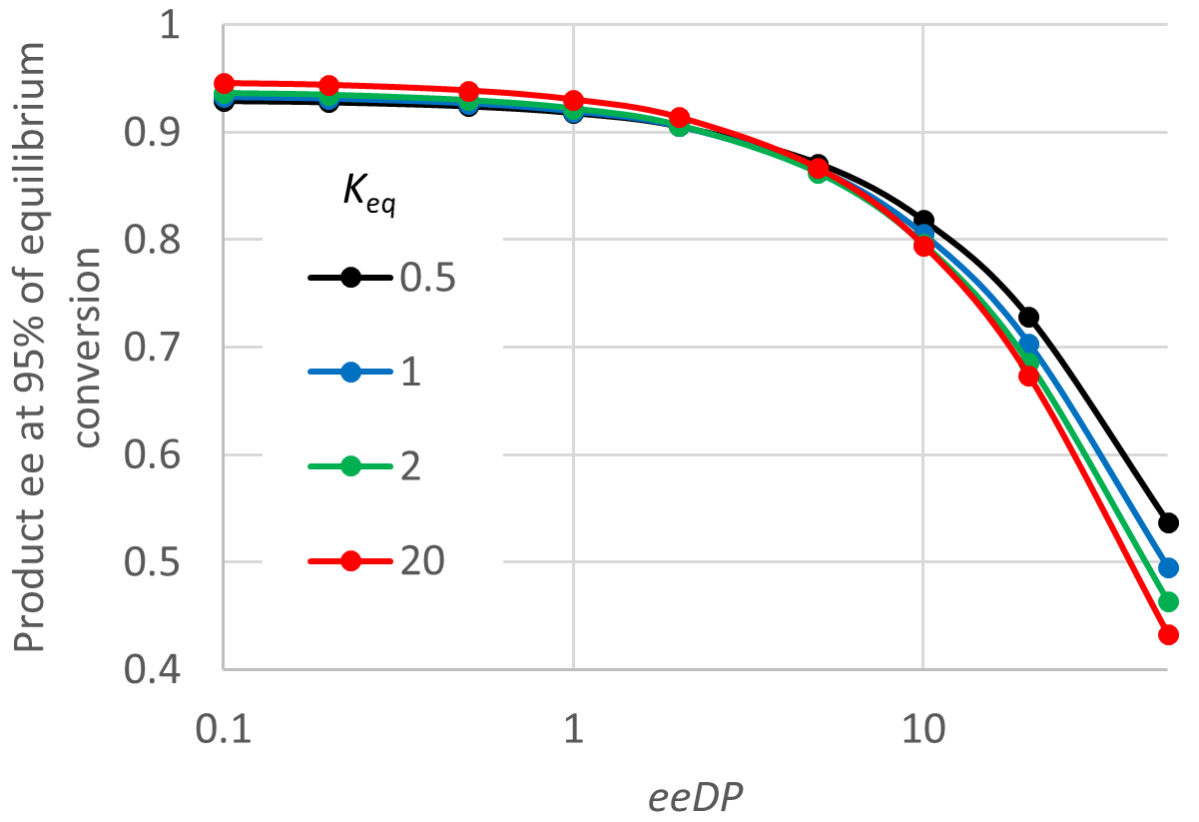
Effect of the key ee decline parameter (*eeDP*) of the enzyme on the product ee for “ping-pong, second” kinetics. *S*_0_ = 0.2 M, D = 0.4 M, *E* = 39, *K*_MS_ = 0.01 M, *k*_2_ = 1000 s^−1^, *SCRf*/*b* = 1, *k*_2_/*k*_4R_ = 1, *E · k*_4S_/*k*_4R_ = 1, *k*_−1_/*k*_2_ = 1, *k*_3R_/*k*_−4R_ = 1, *k*_3S_/*k*_−4S_ = 1. For this plot, *eeDP* was varied by changing *K*_MD_. But the ee at 95% of equilibrium conversion is almost exactly the same if *eeDP* is varied by changing *K*_MS_ (0.001 to 10 M) or *SCRf*/*b* (0.1 to 10).

Now consider the effects of the enzyme properties on the product ee at 95% of equilibrium conversion. It turns out that almost the entire effect (for a given *E* value) can be accounted for by the value of a single group of input parameters, *K*_MD_ divided by *K*_MS_·*SCRf*/*b*. So again this can be termed an “ee decline parameter (*eeDP*)”. Figure 5 shows that for small values of *eeDP*, the product ee remains fairly close to the initial value of 0.95. But for a *eeDP* greater than 1, and particularly greater than 10, the product ee close to equilibrium declines substantially, often to values that are of little preparative value. For a fixed *eeDP*, Figure 5 shows a much weaker effect of *K*_eq_, and for high values of *eeDP*, increasing *K*_eq_ actually worsens the decline in the product ee (for fixed *eeDP*). At a low *eeDP*, increasing *K*_eq_ improves the product ee close to equilibrium, getting nearer the initial value of 0.95 (0.946 for *eeDP* = 0.1 and *K*_eq_ = 20).

From the analytical expression for the initial rate it is found that:


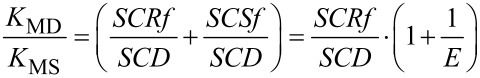


and hence we can write *eeDP* as:


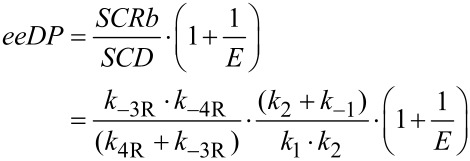


The ratio of the pseudo-specificity constants here governs the competition between the product PR and the donor D in reacting with the free enzyme. The relative rates will be given by *SCRb · PR* divided by *SCD · D*. It makes sense that a large value of this ratio will lead to a faster decline in ee as the reaction approaches equilibrium. There will be some tendency for the *eeDP* to be larger for smaller values of *K*_eq_, because of backwards rate constants in the numerator and forward ones in the denominator. However, there will not be a fixed relationship, as a change in *K*_eq_ may actually be reflected in other rate constants. With the input parameter definitions used here, changes in *K*_eq_ are largely accommodated by changes in *k*_−2_, and a given enzyme may adjust to changes in *K*_eq_ in many other ways.

A list of other enzyme parameters was found to have no noticeable effect on the product ee at 95% of equilibrium conversion: k_2_, *k*_2_/*k*_4R_, *E · k*_4S_/*k*_4R_, *k*_−1_/*k_2_*, *k*_3R_/*k*_−4R_, *k*_3S_/*k*_−4S_.

### Behaviour for “ping-pong, first” kinetics

**Figure 6:**
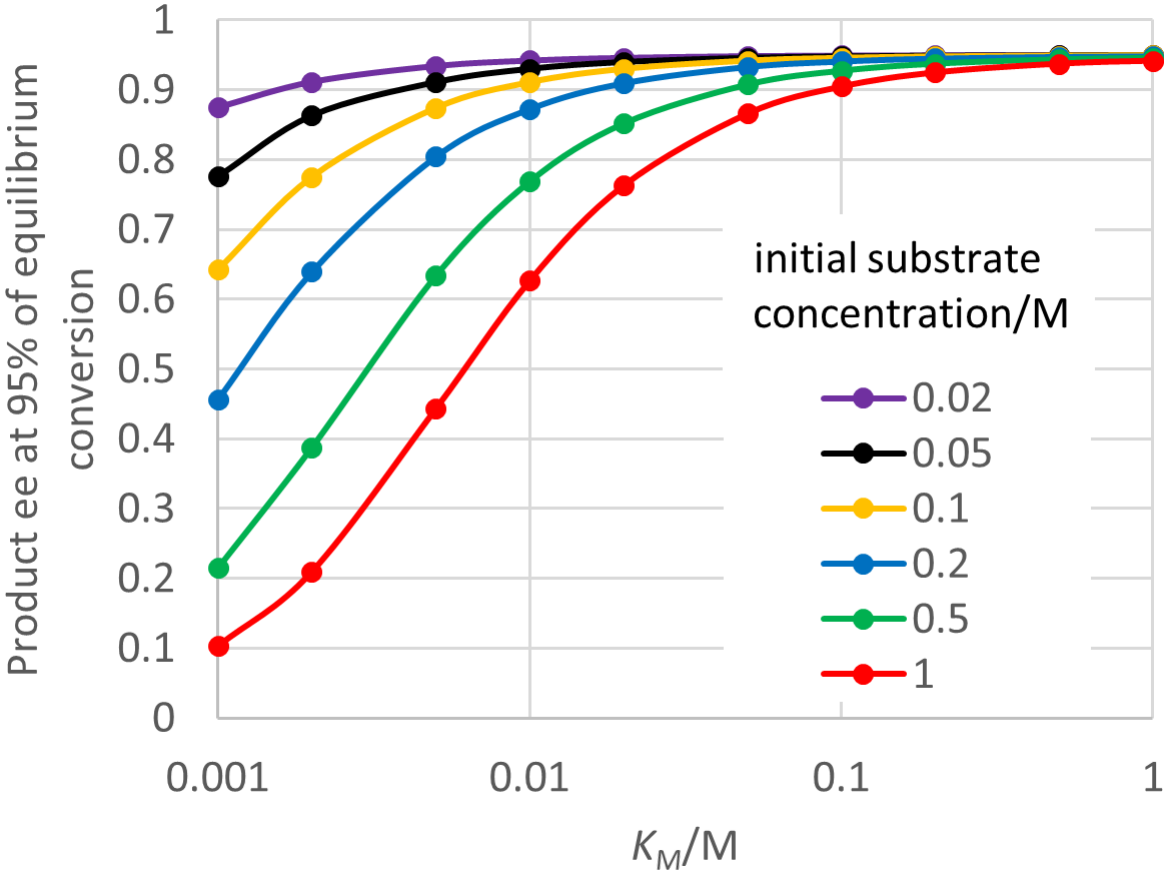
Effects of prochiral substrate concentration and its *K*_M_ value for “ping-pong, first” kinetics. Input parameters were: *K*_eq_ = 15 M, *E* = 39, *k*_4_ = 1000 s^−1^, *SCRf*/*b* = 5, *k*_2R_/*k*_4_ = 1, *k*_2R_/*E · k*_2S_ = 1, *k*_3_ · [H_2_O]/*k*_4_ = 1, *k*_−4_ · *K*_eq_/*k*_4_* =* 1*, k*_−1R_/*k*_−3_ = 1, *E · k*_−1S_/*k*_−3_ = 1. The *K*_eq_ here is defined omitting the water concentration, so has units. For a diol ester hydrolysis, *K*_eq_ will probably depend significantly on the positions of the hydroxy groups with respect to each other. A value around 40 M was found for glycerol dioctanoate [35].

This model will normally apply in enzymatic hydrolysis of prochiral diol esters, so simulations were run for the case where the second substrate (H_2_O in this case) is in constant excess. As can be seen from Figure 6, the prochiral substrate concentration *S*_0_ has a major effect on whether the product ee falls substantially below the initial value. For a low initial substrate concentration or high *K*_M_, the ee remains close to its initial value of 0.95. However, for plausible lower *K*_M_ values, and preparatively more attractive substrate concentrations, the product ee obtained on approach to equilibrium can be greatly reduced. In fact, the value of the product ee is similar for the same value of *S*_0_/*K*_M_, regardless of the individual values. It begins to fall noticeably for *S*_0_/*K*_M_ > 1, and is greatly depressed for *S*_0_/*K*_M_ > 20. Note again that a low *K*_M_ value for the prochiral substrate would normally be seen as a good characteristic of the enzyme, but in fact may lead to problems with a declining ee at high conversion.

**Figure 7:**
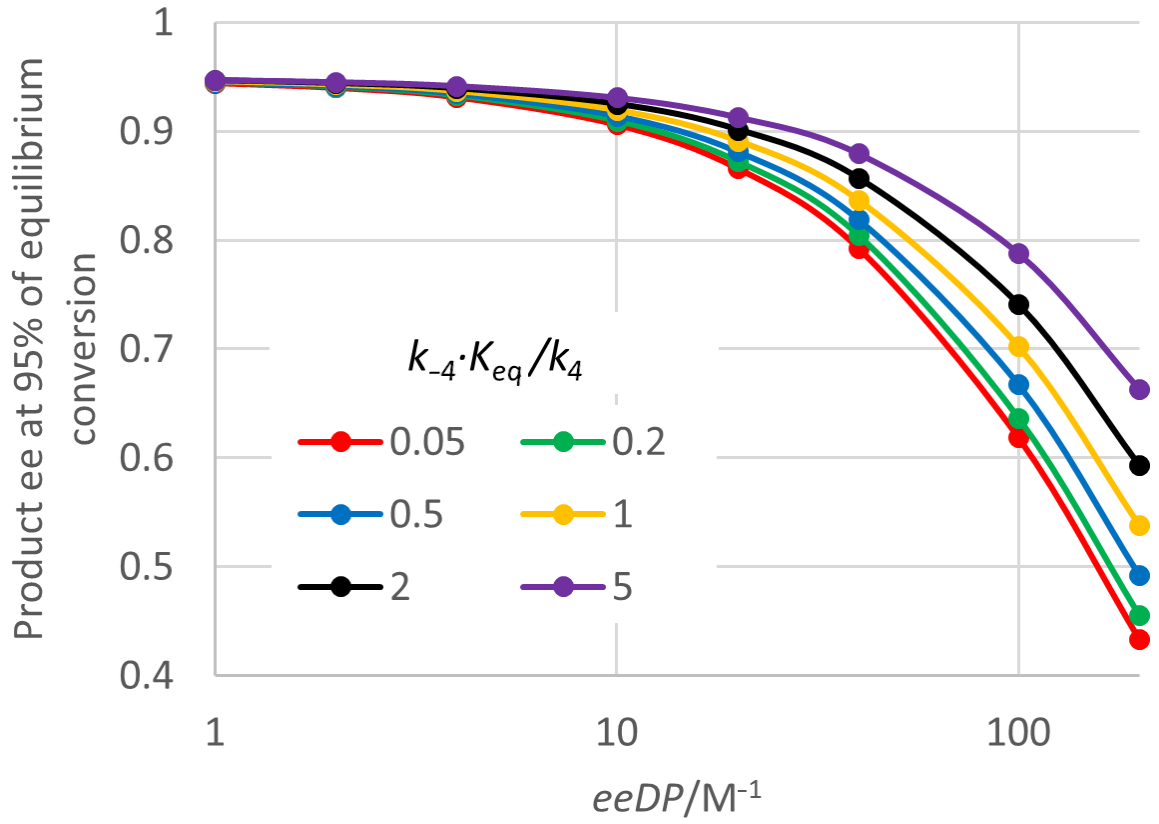
Effect of *eeDP* and *k*_−4_ · K_eq_/*k*_4_ on the product ee at high conversion for “ping-pong, first” kinetics. *S*_0_ = 0.2 M, *K*_eq_ = 15 M, *E* = 39, *k*_4_ = 1000 s^−1^, *SCRf*/*b* = 5, *k*_2R_/*k*_4_ = 1, *k*_2R_/*E · k*_2S_ = 1, *k*_3 _*·* [H_2_O]/*k*_4_ = 1, *k*_−1R_/*k*_−3_ = 1, *E · k*_−1S_/*k*_−3_ = 1. For this plot, different values of *eeDP* (= 1/(*K*_M_ · *SCRf*/*b*)) were obtained by varying *K*_M_, but similar product ee values were found by varying *SCRf*/*b* instead.

Focussing on enzyme properties, it is again possible to identify a single ee decline parameter (*eeDP*) that accounts for the major effects on the fall in the product ee as the reaction proceeds. As Figure 6 would suggest, one component of this is the *K*_M_ value, but the *eeDP* here is defined as the reciprocal of *K*_M_ times *SCRf*/*b*. However, in this case other enzyme parameters were found to have a noticeable, although smaller, effect on the fall in the ee. Figure 7 shows the effect of *eeDP* and also the next most influential parameter *k*_−4_ · *K*_eq_/*k*_4_. For an *eeDP* of 1 M^−1^ or less, the product ee remains close to the initial value of 0.95 throughout. However, for a *eeDP* greater than 10 M^−1^ the product ee can be substantially reduced. For a given value of *eeDP*, the reduction in the product ee at high conversion is less for a larger value of *k*_−4_ · *K*_eq_/*k*_4_, that is when the reaction of the enzyme with the acid byproduct is more kinetically favoured. A referee has pointed out that the product ee at high conversion might be improved by adding a nucleophile that deacylates E* better than water does. The full analysis of the kinetics shows that the *K*_M_ value is actually given by *SCRf* multiplied by a rather complicated function of other rate constants (see Supporting Information File 1). Hence the *eeDP* is actually equal to *SCRb* times this function of other rate constants. It is intuitively reasonable that there should be an important role for this pseudo-specificity constant, which applies to the reaction of the favoured enantiomer product with the acyl enzyme to regenerate the prochiral diol starting material. Three other parameters had small effects on the decline in product ee at high conversion: *k*_2R_/*k*_4_, *k*_2R_/*E · k*_2S_ and *K*_eq_. Four others were found to have no detectable effect: *k*_4_, *k*_3 _*·* [H_2_O]/*k*_4_, *k*_−1R_/*k*_−3_ and *E · k*_−1S_/*k*_−3_.

### Behaviour for “ping-pong, both” kinetics

This mechanism will be found for reactions of prochiral diacids using most lipases and esterases: either in the hydrolysis of their diesters, or the synthesis of a monoester from the free acid. Because this kinetic mechanism has different reactions for the two enantiomers at every stage, there are no less than 16 elementary rate constants involved. The same model applies to both hydrolysis and esterification reactions, but because the initial reactant concentrations are very different in the two cases, the behaviour is discussed separately.

In the case of the diester hydrolysis, the product Q is an alcohol and the reactant B is water, normally present at a high concentration such that it hardly changes as the reaction proceeds. For such reactions, the conclusion from the simulations is relatively simple. There seem to be no plausible enzyme parameters for which the product ee drops substantially below the value found at the start of the reaction. For an *E* value of 39, such that the initial product has an ee of 0.95, it did not drop below 0.919 at 95% of equilibrium conversion for any combinations of parameters tested.

**Figure 8:**
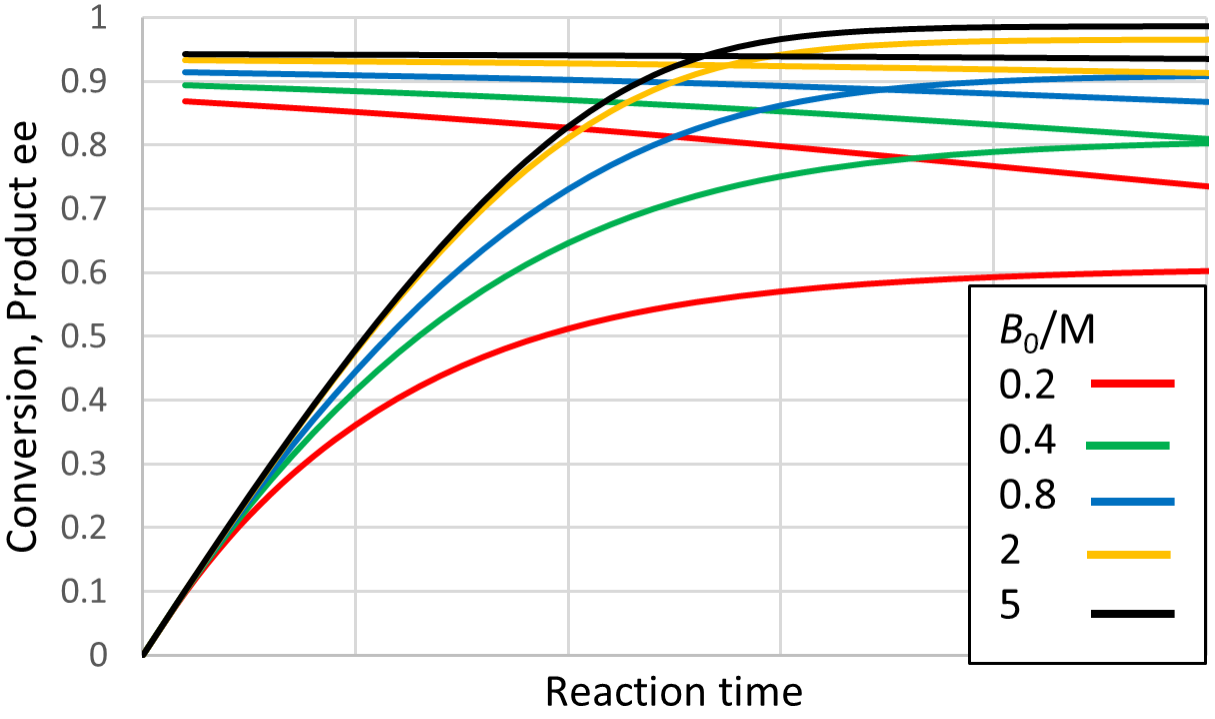
Progress curves for “ping-pong, both” kinetics, diacid esterification. The plot shows the increasing conversion of S and decreasing ee of the product. Parameters not changed: *S*_0_ = 0.2 M, *Q*_0_ = 0.5 M, *K*_eq_ = 10, *E* = 39, *K*_S_ = 0.1 M, *k*_2R_ = 1000 s^−1^, *SCRf*/*b* = 0.5, *E2*/*E* = 0.5, *SCR2f*/*SCRf* = 1, *SCSf*/*b* = 1, *k*_4R_/*k*_2R_ = *k*_2R_/*E · k*_2S_ = *k*_4R_/*E · k*_4S_ = 1, *k*_−1R_/*k*_2R_ = *k*_−1S_/*k*_2S_ = *k*_−3R_/*k*_4R_ = *k*_−3S_/*k*_4S_ = 2. There seems to be no data on *K*_eq_ for diacid ester reactions, but the values range between 2 and 4 for a simple esterification in various non-aqueous solvents [35].

**Figure 9:**
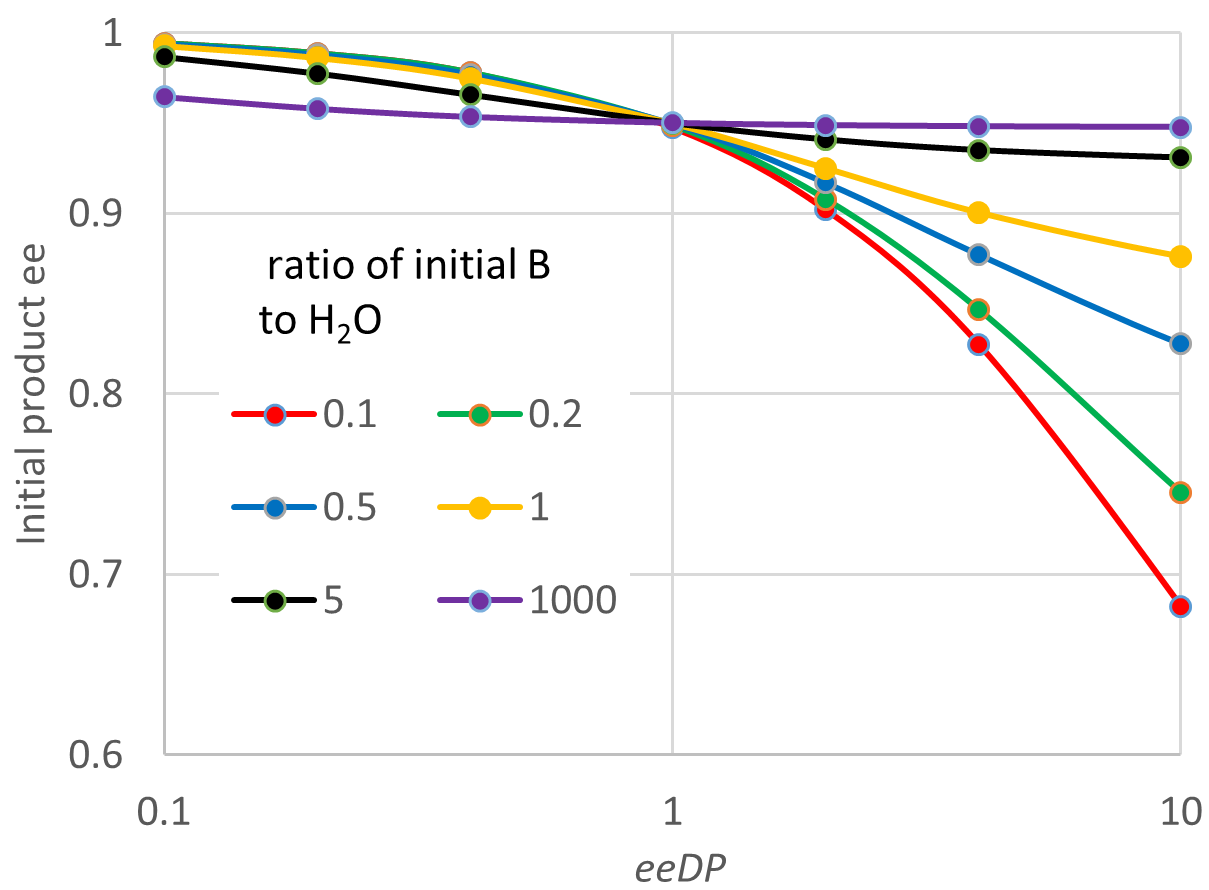
Effects on the ee of the product formed early in the reaction for “ping-pong, both” kinetics, diacid esterification. The graph plots ee at a conversion of about 0.1. For this plot the value of *eeDP* was altered by varying *SCSf*/*b*, but very similar ee values were found if *SCRf*/*b* or *E2*/*E* were varied instead. Similarly, *Q*_0_ (the initial H_2_O concentration) was varied for the plot, but the ratio *B*_0_/*Q*_0_ is what actually determines the behaviour. Parameters not varied were: *S*_0_ = 0.2 M, *B*_0_ = 0.4 M, *K*_eq_ = 10, *E* = 39, *K*_S_ = 0.1 M, *k*_2R_ = 1000 s^−1^, *SCRf*/*b* = 0.5, *E2*/*E* = 0.5, *SCR2f*/*SCRf* = 1, *k*_4R_/*k*_2R_ = *k*_2R_/*E · k*_2S_ = *k*_4R_/*E · k*_4S_ = 1, *k*_−1R_/*k*_2R_ = *k*_−1S_/*k*_2S_ = *k*_−3R_/*k*_4R_ = *k*_−3S_/*k*_4S_ = 2.

**Figure 10:**
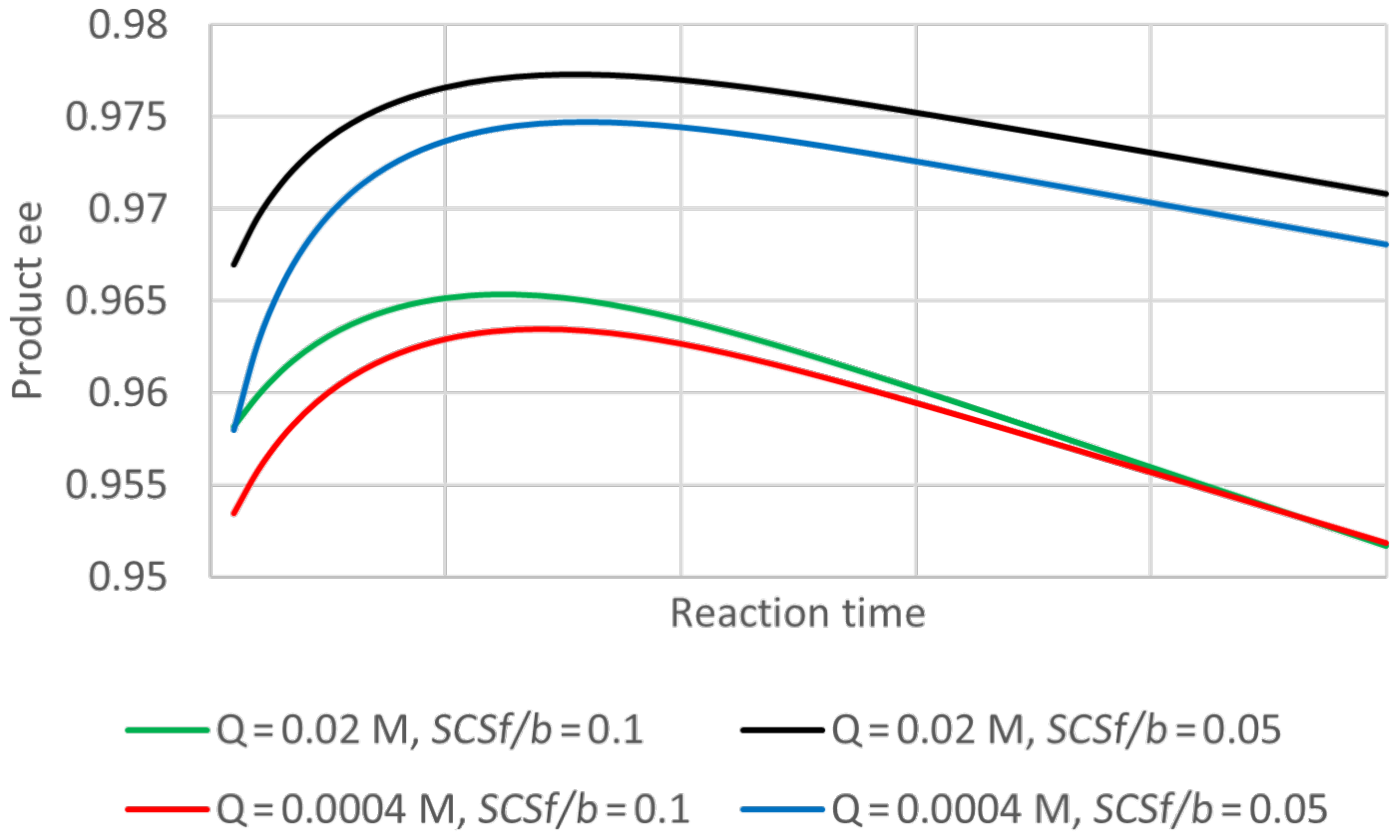
Increase in the product ee as the reaction proceeds for “ping-pong, both” kinetics, diacid esterification. There is no point for exactly zero time, because no product has been formed. Other parameters were: *S*_0_ = 0.2 M, *B*_0_ = 0.5 M, *K*_eq_ = 10, *E* = 39, *K*_S_ = 0.1 M, *k*_2R_ = 1000 s^−1^, *SCRf*/*b* = 0.5, *E2*/*E* = 0.5, *SCR2f*/*SCRf* = 1, *k*_4R_/*k*_2R_ = *k*_2R_/*E · k*_2S_ = *k*_4R_/*E · k*_4S_ = 1, *k*_−1R_/*k*_2R_ = *k*_−1S_/*k*_2S_ = *k*_−3R_/*k*_4R_ = *k*_−3S_/*k*_4S_ = 2. By the end of the reaction time plotted, the conversion is about 0.92, very close to the equilibrium value.

In the case of an esterification, the product Q is water, and will normally be present at a substantial initial concentration (even though a non-aqueous medium is usual). The reactant B will be an alcohol. Figure 8 shows some progress curves for plausible conditions and enzyme parameters under which the product ee deviates noticeably from 0.95 expected for the value of *E*. In this case even the initial product ee differs from 0.95 except when the concentration of B is high. This is surprising behaviour, but its occurrence was also supported by the analytical solution of the quasi-steady state equations. These solutions suggest that the key parameter is actually the ratio of B to Q (i.e., B to H_2_O). Figure 9 shows how the initial ee depends on the ratio *B*_0_/*Q*_0_, and the most influential enzyme parameter, which can again be termed ee decline parameter (*eeDP*). In this case the empirical analysis showed that this parameter was *E · SCSf*/*b* divided by (*E2 · SCRf*/*b*). Note, how for small values of *eeDP* and *B*_0_/*Q*_0_, the product ee can be greater than the value of 0.95 expected from the *E* of 39. Hence for this type of reaction, a single value of ee does not give general information about even the initial performance of the enzyme. An even more surprising observation for this type of reaction is illustrated in Figure 10, where the product ee can actually increase as the reaction proceeds, at least at first. This is observed consistently for somewhat unusual, but not impossible, circumstances with very low concentrations of Q (H_2_O) and small values of the enzyme parameter *SCSf*/*b*.

**Figure 11:**
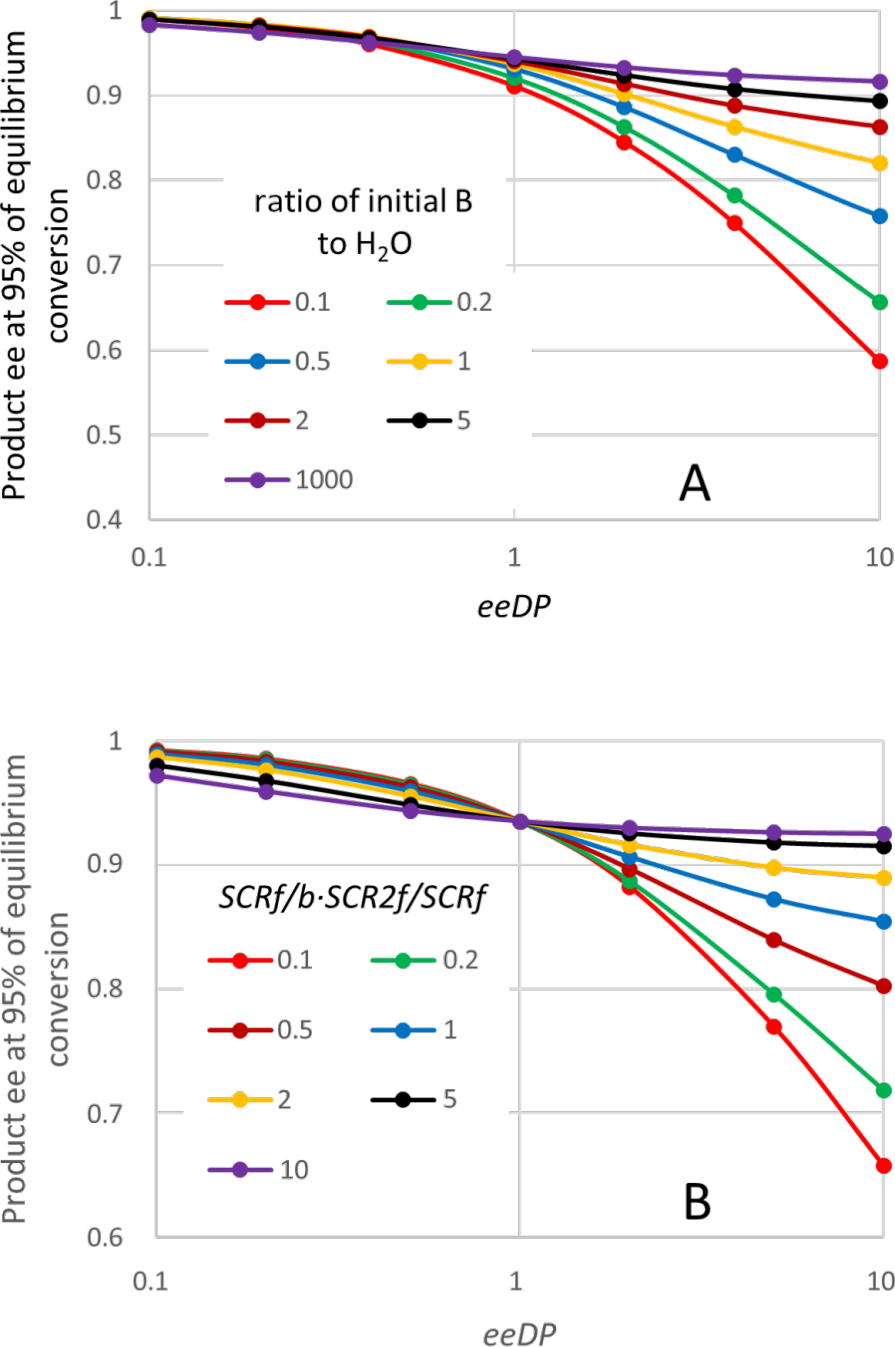
Effects on the ee at high conversion for diacid ester synthesis, “ping-pong, both” kinetics. Parameters not varied were: *S*_0_ = 0.2 M, *B*_0_ = 0.4 M, *K*_eq_ = 10, *E* = 39, *K*_S_ = 0.1 M, *k*_2R_ = 1000 s^−1^, *SCR2f*/*SCRf* = 1, *k*_4R_/*k*_2R_ = *k*_2R_/*E · k*_2S_ = *k*_4R_/*E · k*_4S_ = 1, *k*_−1R_/*k*_2R_ = *k*_−1S_/*k*_2S_ = *k*_−3R_/*k*_4R_ = *k*_−3S_/*k*_4S_ = 2. For part A, the ratio *B*_0_/*Q*_0_ was varied by changing *Q*_0_ (H_2_O), although almost identical product ees were obtained for the same ratio, if *B*_0_ was varied instead. Similarly, the ee decline parameter (*eeDP*) was varied by changing *SCSf*/*b*, with *SCRf*/*b* = 0.5 and *E2*/*E* = 0.5, but changing these two parameters gave similar product ees for any given value of *eeDP*. For part B, *Q*_0_ = 0.5 M, *SCSf*/*b* = 1. The parameters *SCRf*/*b* and *E2*/*E* were both varied, so that each value of *eeDP* could be obtained with different combinations of *SCRf*/*b* and *E2*/*E*. Each point would have a different value of the product *SCRf*/*b · SCR2f*/*SCRf*. If instead *SCR2f*/*SCRf* was varied, but the product and *eeDP* were kept constant, the ee values found for the reaction product were very similar.

Figure 11 shows the effects on the product ee at 95% of the equilibrium conversion, as for the other kinetic mechanisms. Again these ee values can deviate substantially from 0.95 that might be expected for the *E* value of 39 used. And for some parameters, they can actually exceed 0.95, as seen already in the initial rate. The general shape of the plots in part A are similar to those for the initial ee in Figure 9. The plot is again against the ee decline parameter (*eeDP*), which is the enzyme property with the most important influence on the product ee. However, as shown in part B, in this case there remain important effects of other parameters. An empirical study showed that most of the remaining effect could be accounted for by the value of the product of parameters *SCRf*/*b* and *SCR2f*/*SCRf*. For *eeDP* > 1, lower values of *SCRf*/*b · SCR2f*/*SCRf* cause a much larger fall in the ee as the reaction approaches equilibrium. In contrast, for *eeDP* < 1, lower values of *SCRf*/*b · SCR2f*/*SCRf* actually lead to a higher product ee, often greater than 0.95 expected from the *E* of 39. Parameters not varied in Figure 11 had little or no effect on the product ee. It decreased slightly for a higher *S*_0_, and increased slightly for a higher *K*_eq_. There was no noticeable effect of varying *K*_S_, *k*_2R_, *k*_4R_/*k*_2R_, *k*_2R_/*E · k*_2S_, *k*_4R_/*E · k*_4S_, *k*_−1R_/*k*_2R_, *k*_−1S_/*k_2S_*, *k*_−3R_/*k*_4R_ or *k*_−3S_/*k*_4S_.

Expanding the parameters incorporated in *eeDP* and making some cancellations leads to


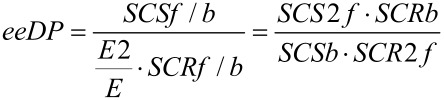


The right hand expression has two interesting ratios of pseudo-specificity constants. *SCS2f*/*SCSb* governs the relative rate of the reaction of the S acyl enzyme with either B to produce S ester product or with Q (H_2_O) to give back the prochiral diacid. *SCR2f*/*SCRb* acts the same way for the R acyl enzyme. It makes intuitive sense that such ratios would be important, as the formation of the unwanted S product will be favoured, if the S acyl enzyme tends to react forward or the R acyl enzyme tends to react backwards. A similar expansion and cancellation from the empirically found product *SCRf*/*b · SCR2f*/*SCRf* shows that this is also equal to *SCR2f*/*SCRb*.

In summary, for this case of diacid ester synthesis, reporting a single product ee as characterising the reaction is particularly misleading. Not only can it vary as the reaction proceeds, but even the initial value can change, including as a result of experimental variables like the initial B (alcohol) concentration.

## Conclusion

The kinetics of enzymatic desymmetrisation of prochiral starting materials is not as simple as usually assumed. It is a mistake to assume that the product ee will always remain constant throughout the progress of the reaction. The kinetics of many enzymes may lead to a product ee declining substantially before reaching preparatively useful conversions. Hence when choosing or modifying enzymes for these reactions it is important to consider the extent to which the ee might fall over the time course, as well as its initial value. For the various kinetic mechanisms, it is possible to identify a single enzyme property, an ee decline parameter (*eeDP*), that describes the dominant tendency.

The work indicates that good practice in reporting enzymatic desymmetrisation experiments would be to state conversion at which an ee value is found. Preferably at least two pairs of conversion and ee values should be reported for a given reaction, to show how much the ee tends to decline at higher conversion.

Some effects found could lead to problems in improving a preparative reaction based on initial studies. Clearly a high ee found in initial work at low conversion may be found to decline substantially when the reaction time is extended to reach preparative conversions. But the adverse effect (for some kinetic mechanisms) of higher concentrations of the prochiral starting material should also be noted. Very often process development will involve increasing these concentrations to try and obtain a more efficient process, but the result may be an undesirable fall in ee at higher conversions.

## Supporting Information

A single file of Supporting Information is available. It contains the Methods section, with full details of how simulations were performed and the mathematical analysis of the various kinetic mechanisms. It also gives a fuller index of data files (Maple worksheets for the full derivations; MATLAB code files to run the simulations; Excel files containing all calculated progress curves) that can be downloaded via: https://doi.org/10.15129/fbd7e7c0-9712-41a4-a88d-63eecccdd2d9.

File 1 Methods, full details of simulations, and mathematical analysis of kinetic mechanisms. Index of data files and download link.
