# Supplementary material for: Kinetics of enzyme-catalysed desymmetrisation of prochiral substrates: product enantiomeric excess is not always constant
Source: Beilstein J Org Chem. 2021 Apr 21;17:873–84. doi: 10.3762/bjoc.17.73 (PMC8077619; doi:10.3762/bjoc.17.73)
Supplement: File 1 — Methods, full details of simulations, and mathematical analysis of kinetic mechanisms. Index of data files and download link. [file Beilstein_J_Org_Chem-17-873-s001.pdf]

BEILSTEIN JOURNAL OF ORGANIC CHEMISTRY

Supporting Information

for

Kinetics of enzyme-catalysed desymmetrisation of prochiral substrates: product enantiomeric excess is not always constant

Peter J. Halling

Beilstein J. Org. Chem. **2021,** *17,* 873–884. doi:10.3762/bjoc.17.73

Methods, full details of simulations, and mathematical analysis of kinetic mechanisms. Index of data files and download link

License and Terms: This is a supporting information file under the terms of the Creative Commons Attribution License (https://creativecommons.org/licenses/by/4.0). Please note that the reuse, redistribution and reproduction in particular requires that the author(s) and source are credited and that individual graphics may be subject to special legal provisions.
The license is subject to the *Beilstein Journal of Organic Chemistry* terms and conditions: (https://www.beilstein-journals.org/bjoc/terms)

Index to data files available for download

All the following are freely available for download via this link:

https://doi.org/10.15129/fbd7e7c0-9712-41a4-a88d-63eecccdd2d9

Maple worksheets for each of the 4 kinetic models, both in native (.mw) form, and as pdf print-outs.

ProchiralOrdered.mw

ProchiralPing.mw (for “ping-pong, second” kinetics)

ProchiralDiolEsters.mw (for “ping-pong, first” kinetics)

ProchiralDiacids.mw (for “ping-pong, both” kinetics)

MATLAB code files for each of the kinetic models (two for the Diacid case, covering hydrolysis and esterification).

enz_progress_ProchiralOrdered.m

enz_progress_ProchiralPing.m

enz_progress_ProchiralDiol.m

enz_progress_ProchiralDiacid_Hydr.m

enz_progress_ProchiralDiacid_Estfn.m

which call functions defined in separate files:-

ODE_ProchiralOrdered.m

ODE_ProchiralPing.m

ODE_ProchiralDiol.m

ODE_ProchiralDiacid.m

Excel files with full simulated progress data for a wide range of input parameter values.

PlotGraphsOrdered.xlsm

PlotGraphsPing.xlsm

PlotGraphsDiol.xlsm

PlotGraphsDiacid.xlsm

METHODS

Calculation of reaction progress curves by numerical method

Based on the kinetic mechanisms shown in Scheme 1, ordinary differential equations were written for the change in concentration of all enzyme forms, reactants, and products. This set of equations was then integrated using MATLAB, based on the elementary rate constants, and no analytical approximations. The in-built ode15s solver was able to handle the integration reasonably quickly (usually less than 10 s on a 7 years old laptop), despite the equation system being stiff. (Avoiding a stiff equation set is one reason for

the usual approach of using the quasi-steady state approximation). The tolerance was routinely set at 1E-6; changing to 1E-5 or 1E-7 gave the same results to at least 1 part in 1000. The differential equations were actually handled in normalised form without dimensions of concentration:

all enzyme forms were handled as fractions of the total enzyme concentration (E_0);

S, D, B, Q, PR, and PS were all normalised via division by the initial substrate concentration S_0;

and all second order rate constants were multiplied by S_0 to give values with dimensions of time^{-1}.

The ratio of E_0/S_0, typically a small number, appeared as a multiplier for many coefficients in the differential equations. So, for example the fully dimensioned equation:

$$\frac{dS}{dt} = -k_{2R}.ED.S - k_{2S}.ED.S + k_{-2R}.EDS_R + k_{-2S}.EDS_S$$

for the ordered kinetic model is transformed to the normalised form:

$$\frac{dS'}{dt} = \frac{E_0}{S_0}.(-k'_{2R}.ED'.S' - k'_{2S}.ED'.S' + k_{-2R}.EDS_R' + k_{-2S}.EDS_S')$$

where apostrophes (‘) indicate normalised values. Using these normalised forms has the effect that all state variables are of a similar magnitude (typically 0.1 to 10) throughout most of the reaction progress, which may help with the integration of the model (although this was not tested).

To aid comparison of progress curves for different enzyme properties and initial concentrations, the ratio E_0/S_0 was set so that the initial rate was always the same. This is essentially equivalent to using a dimensionless time value. Hence no times are given on the progress curves shown. The necessary value of E_0/S_0 was calculated using an expression for the initial rate obtained through the mathematical analysis of the kinetics (see below).

Driver MATLAB code took values of the appropriate input parameters (see below), calculated the corresponding elementary rate constants, ran the integration, then output values of conversion (total to oth product enantiomers) and enantiomeric excess for a series of time points.

All the MATLAB code files used, and all the progress data generated, are freely available for download via the link given at the top of this document.

Mathematical analysis of kinetics

As an aid to understanding, and the selection of sensible input enzyme parameters, equations were also solved analytically following the quasi-steady state approximation (setting the rate of change of all enzyme forms to zero). After elimination from the resulting equations (using Maple software to deal with some very

large and complicated expressions), it was possible to derive equations for the rate of product formation. Based on this analysis, parameters were selected as inputs for the calculation of progress curves, following these principles:

a) values for which separate estimates will often be possible, like K_{eq} and E, the observed ratio of enantiomers (under initial rate conditions);

b) inspection of the analytical equations for reaction progress, derived using the quasi-steady state approximation;

and c) empirical observations of highly influential parameters after trials with different possible sets that might be used.

These input parameters were then used to calculate the elementary rate constants used in calculation of progress curves.

The mathematical analysis is now summarised for each kinetic mechanism as defined in Scheme 1 of the main paper, using elementary rate constants for reaction steps shown in that Scheme. The links at the top of this document allow download of all the Maple worksheets, and pdf print-outs of them.

"Ordered, second" kinetics

The set of linked differential equations were solved analytically using the pseudo-steady state approximation that sets the rate of change of enzyme forms to zero. One of the most useful forms for the overall rate equation obtained is the following:

$$\frac{1}{E_0}\frac{dPR}{dt} =$$

$$\frac{\frac{D.S.k_1.k_4.k_{2R}.k_{3R}}{k_{3R}+k_{-2R}} + \frac{D.S.PS.k_1.k_{2R}.k_{3R}.k_{-2S}.k_{-3S}}{(k_{3R}+k_{-2R}).(k_{3S}+k_{-2S})} + \frac{S.Q.PS.k_{2R}.k_{3R}.k_{-2S}.k_{-3S}.k_{-4}}{(k_{3R}+k_{-2R}).(k_{3S}+k_{-2S})} - \frac{PR.Q.k_{-1}.k_{-4}.k_{-2R}.k_{-3R}}{k_{3R}+k_{-2R}} - \frac{PR.D.S.k_1.k_{2S}.k_{3S}.k_{-2R}.k_{-3R}}{(k_{3R}+k_{-2R}).(k_{3S}+k_{-2S})} - \frac{PR.S.Q.k_{2S}.k_{3S}.k_{-2R}.k_{-3R}.k_{-4}}{(k_{3R}+k_{-2R}).(k_{3S}+k_{-2S})}}{\begin{array}{l} k_4.k_{-1} + D.k_1.k_4 + Q.k_{-1}.k_{-4} + S.k_4.\left(\frac{k_{2R}.k_{3R}}{(k_{3R}+k_{-2R})} + \frac{k_{2S}.k_{3S}}{(k_{3S}+k_{-2S})}\right) + \frac{PR.k_{-1}.k_{-2R}.k_{-3R}}{k_{3R}+k_{-2R}} \\ + \frac{PS.k_{-1}.k_{-2S}.k_{-3S}}{k_{3S}+k_{-2S}} + D.S.k_1.\left(\frac{k_{2R}.(k_{3R}+k_4)}{(k_{3R}+k_{-2R})} + \frac{k_{2S}.(k_{3S}+k_4)}{(k_{3S}+k_{-2S})}\right) + \frac{D.PR.k_1.k_{-2R}.k_{-3R}}{k_{3R}+k_{-2R}} \\ + \frac{D.PS.k_1.k_{-2S}.k_{-3S}}{k_{3S}+k_{-2S}} + S.Q.k_{-4}.\left(\frac{k_{2R}.k_{3R}}{(k_{3R}+k_{-2R})} + \frac{k_{2S}.k_{3S}}{(k_{3S}+k_{-2S})}\right) + \frac{Q.PR.k_{-4}.k_{-3R}.(k_{-1}+k_{-2R})}{(k_{3R}+k_{-2R})} \\ + \frac{Q.PS.k_{-4}.k_{-3S}.(k_{-1}+k_{-2S})}{(k_{3S}+k_{-2S})} + D.S.PR.k_1.k_{-3R}.\left(\frac{k_{2S}.(k_{3S}+k_{-2R}) + k_{2R}.(k_{3S}+k_{-2S})}{(k_{3R}+k_{-2R}).(k_{3S}+k_{-2S})}\right) \\ + D.S.PS.k_1.k_{-3S}.\left(\frac{k_{2R}.(k_{3R}+k_{-2S}) + k_{2S}.(k_{3R}+k_{-2R})}{(k_{3R}+k_{-2R}).(k_{3S}+k_{-2S})}\right) \\ + S.Q.PR.k_{-4}.k_{-3R}.\left(\frac{k_{2S}.(k_{3S}+k_{-2R}) + k_{2R}.(k_{3S}+k_{-2S})}{(k_{3R}+k_{-2R}).(k_{3S}+k_{-2S})}\right) \\ + S.Q.PS.k_{-4}.k_{-3S}.\left(\frac{k_{2R}.(k_{3R}+k_{-2S}) + k_{2S}.(k_{3R}+k_{-2R})}{(k_{3R}+k_{-2R}).(k_{3S}+k_{-2S})}\right) \end{array}}$$

Since the derivation makes no assumption about whether the R or S enantiomer is favoured, the corresponding expression for dPS/dt is simply obtained by exchanging every R for S and vice versa in this expression. In fact, the appearances of R and S in the denominator are completely symmetrical, so the denominator does not change with this substitution. The expression contains multiple appearances of the sums ($k_{3R} + k_{-2R}$) and ($k_{3S} + k_{-2S}$), which arrive naturally from the derivation. Furthermore ($k_{3R} + k_{-2R}$) often appears as the denominator in terms with products $k_{2R}{\cdot}k_{3R}$ or $k_{-2R}{\cdot}k_{-3R}$ in the numerator, as do the corresponding groups in S. The ratio $k_{2R}{\cdot}k_{3R}/(k_{3R} + k_{-2R})$ has the form of a pseudo-specificity constant (k_{cat}/K_M) for the reaction of the enzyme form ED with S to produce PR and EQ. Similarly, $k_{2S}{\cdot}k_{3S}/(k_{3S} + k_{-2S})$ is a pseudo-specificity constant for the corresponding reaction producing PS. These pseudo-specificity constants will govern the competition between these two reaction pathways, and hence the ratio of product enantiomers formed. This can also be seen by considering the full expression above under initial rate conditions, when both PR and PS are zero. The ratio of initial rates of PR and PS formation becomes the ratio of the first terms in the numerator for R and S. And this ratio is exactly the ratio of pseudo-specificity constants. The ratios with backward rate constants in the numerator, like $k_{-2R}{\cdot}k_{-3R}/(k_{3R} + k_{-2R})$, can also be seen as pseudo-specificity constants for the reverse reaction, here of the enzyme form EQ with PR to produce ED and give back S. Hence in understanding the behaviour and simplifying the expression it is useful to define these pseudo-specificity constants as parameters, labelled SC followed by either R or S, then f or b for the forwards or backwards direction.

$$SCRf = \frac{k_{2R}.k_{3R}}{k_{3R}+k_{-2R}}$$

$$SCRb = \frac{k_{-2R}.k_{-3R}}{k_{3R}+k_{-2R}}$$

$$SCSf = \frac{k_{2S}.k_{3S}}{k_{3S}+k_{-2S}}$$

$$SCSb = \frac{k_{-2S}.k_{-3S}}{k_{3S}+k_{-2S}}$$

Substituting these gives the following expression for the rate

$$\frac{dPR}{dt} = E_0.\frac{\begin{array}{c} D.S.k_1.k_4.SCRf + D.S.PS.k_1.SCRf.SCSb + S.Q.PS.k_{-4}.SCRf.SCSb \\ -PR.Q.k_{-1}.k_{-4}.SCRb - PR.D.S.k_1.SCSf.SCRb - PR.S.Q.k_{-4}.SCSf.SCRb \end{array}}{\begin{array}{c} k_4.k_{-1} + D.k_1.k_4 + Q.k_{-1}.k_{-4} + S.k_4.(SCRf + SCSf) + PR.k_{-1}.SCRb \\ +PS.k_{-1}.SCSb + D.S.k_1.\left(SCRf.\left(1+\frac{k_4}{k_{3R}}\right) + SCSf.\left(1+\frac{k_4}{k_{3S}}\right)\right) + D.PR.k_1.SCRb \\ +D.PS.k_1.SCSb + S.Q.k_{-4}.(SCRf + SCSf) + Q.PR.k_{-4}.SCRb.\left(1+\frac{k_{-1}}{k_{-2R}}\right) \\ +Q.PS.k_{-4}.SCSb.\left(1+\frac{k_{-1}}{k_{-2S}}\right) + D.S.PR.k_1.SCRb.\left(\frac{SCSf}{k_{-2R}} + \frac{SCSf}{k_{3S}} + \frac{k_{2R}}{k_{-2R}}\right) \\ +D.S.PS.k_1.SCSb.\left(\frac{SCRf}{k_{-2S}} + \frac{SCRf}{k_{3R}} + \frac{k_{2S}}{k_{-2S}}\right) + S.Q.PR.k_{-4}.SCRb.\left(\frac{SCSf}{k_{-2R}} + \frac{SCSf}{k_{3S}} + \frac{k_{2R}}{k_{-2R}}\right) \\ +S.Q.PS.k_{-4}.SCSb.\left(\frac{SCRf}{k_{-2S}} + \frac{SCRf}{k_{3R}} + \frac{k_{2S}}{k_{-2S}}\right) \end{array}}$$

It should be noted that now k_{-3R} and k_{-3S} no longer appear as separate parameters.

Under initial rate conditions with Q, PS and PR all zero, the expression reduces to

$$\frac{dPR}{dt} = \frac{E_0.D.S.SCRf}{\frac{k_{-1}}{k_1} + D + S.\left(\frac{SCRf + SCSf}{k_1}\right) + D.S.\left(\frac{SCRf}{k_4} + \frac{SCSf}{k_4} + \frac{SCRf}{k_{3R}} + \frac{SCSf}{k_{3S}}\right)}$$

Comparison with the usual expression for the initial rate of an enzyme following the ordered mechanism gives the K_M values for substrates D and S as:

$$K_{MS} = \frac{1}{\left(\frac{SCRf}{k_4} + \frac{SCSf}{k_4} + \frac{SCRf}{k_{3R}} + \frac{SCSf}{k_{3S}}\right)}$$

$$K_{MD} = \frac{\left(\frac{SCRf + SCSf}{k_1}\right)}{\left(\frac{SCRf}{k_4} + \frac{SCSf}{k_4} + \frac{SCRf}{k_{3R}} + \frac{SCSf}{k_{3S}}\right)}$$

In choosing parameters to define the behaviour, it is sensible to include ones that are quite likely to be known or at least be estimated. Hence they include K_{eq} for the overall reaction, and E for the observed ratio of enantiomers (under initial rate conditions). As shown above, E = SCRf/SCSf. Then use K_{MD} and K_{MS}, which will be used in the relationships immediately above. The rate constant arbitrarily fixed to set the overall timescale can be chosen as k_4. Two further parameters selected are the ratios k_{3R}/k_4 (which does appear in the full rate expression) and $k_{3R}/(E\cdot k_{3S})$. Both these parameters are dimensionless, and are expected to be of the order of magnitude of 1 (E is introduced in the definition of the second parameter to bring it closer to 1). Using the values of k_4, k_{3R}/k_4, $k_{3R}/(E\cdot k_{3S})$, E and K_{MS}, the equation above allows calculation of SCRf and SCRb. Then the value of K_{MD} allows calculation of k_1.

The dimensionless ratio SCRf/b = SCRf/SCRb affects the relative magnitude of terms in the full rate expression, and was also found empirically to have a key influence on behaviour. Its introduction allows calculation of SCRb. Now make use of the equilibrium constant for racemisation of the product, which must equal 1, and can be related to SC values, allowing calculation of SCSb:

$$K_{rac} = 1 = \frac{k_{2R}.k_{3R}.k_{-2S}.k_{-3S}.}{k_{2S}.k_{3S}.k_{-2R}.k_{-3R}.} = \frac{SCRf.SCSb}{SCSf.SCRb}$$

Now introduce the ratio k_{-4}/k_1. This also controls the relative magnitude of many terms in the full rate expression; dividing this expression top and bottom by one of these rate constants will generate many examples of k_{-4}/k_1. Setting this ratio as a parameter allows the calculation of k_{-4}. Then make use of the equilibrium constant K_{eq} for formation of PR and Q from D and S, which is related to rate constants by

$$K_{eq} = \frac{k_1.k_{2R}.k_{3R}.k_4}{k_{-1}.k_{-2R}.k_{-3R}.k_{-4}} = \frac{k_1.k_4.SCRf}{k_{-1}.k_{-4}.SCRb}$$

which allows calculation of k_{-1}.

Finally introduce two more ratios k_{-2R}/k_{-1} and $E{\cdot}k_{-2S}/k_{-1}$. Both these ratios of rate constants appear in the full rate expression, and they are both first order rate constants so the ratio is dimensionless. Their values are again likely to be of the order of magnitude of 1. Using k_{-2R}/k_{-1} and $E{\cdot}k_{-2S}/k_{-1}$, the values of k_{-2R} and k_{-2S} can be calculated, and hence all remaining rate constants using the known values of SC's.

To aid comparison of progress simulations, it is desirable that they all have the same initial rate. In the case of ordered kinetics, it is assumed that cofactor recycling will be used, so the conditions are approximated as having constant non-zero concentrations of both D and Q (e.g., NADH and NAD^+). Under these conditions the full rate expression reduces to:

$$\frac{1}{S_0}\frac{dPR}{dt} = \frac{E_0.D.k_1.k_4.SCRf}{\begin{array}{c} k_4.k_{-1} + D.k_1.k_4 + Q.k_{-1}.k_{-4} + S_0.k_4.(SCRf + SCSf) \\ +D.S_0.k_1.\left(SCRf.\left(1+\frac{k_4}{k_{3R}}\right) + SCSf.\left(1+\frac{k_4}{k_{3S}}\right)\right) + S_0.Q.k_{-4}.(SCRf + SCSf) \end{array}}$$

Where S_0 is the initial concentration of S and $(1/S_0){\cdot}(dPR/dt)$ is the dimensionless initial rate of conversion to PR. There will also be an initial rate of conversion to PS, but because of the symmetry in the expressions, this will be a fixed fraction of that to PR, for any given E value. Hence the rate of increase in overall conversion will be higher by a factor of (1 + 1/E). Assuming the R enantiomer is favoured, this factor will be slightly greater than 1; if the S enantiomer is favoured, it is probably best to exchange R and S in the expressions above, such that E is again defined such that it is greater than 1.

"Ping-pong, second" kinetics

In this case the usual pseudo-steady state approximation leads to the following full expression for the rate of product formation:

$$\frac{dPR}{dt} = E_0.\frac{\frac{D.S.k_1.k_2.k_{3R}.k_{4R}}{(k_{4R}+k_{-3R})} + \frac{S.PS.k_{3R}.k_{-3S}.k_{-4S}.(k_2+k_{-1})}{(k_{4S}+k_{-3S})} - \frac{PR.Q.k_{-1}.k_{-2}.k_{-3R}.k_{-4R}}{(k_{4R}+k_{-3R})} - \frac{PR.S.k_{-3R}.(k_2+k_{-1}).(k_{3S}.k_{4S}.k_{-4R}+k_{3R}.k_{-3S}.k_{-4S})}{(k_{4R}+k_{-3R}).(k_{4S}+k_{-3S})}}{D.k_1.k_2 + S.(k_2+k_{-1}).\left(\frac{k_{3R}.k_{4R}}{(k_{4R}+k_{-3R})}+\frac{k_{3S}.k_{4S}}{(k_{4S}+k_{-3S})}\right) + Q.k_{-1}.k_{-2} + \frac{PR.k_{-3R}.k_{-4R}.(k_2+k_{-1})}{(k_{4R}+k_{-3R})} + \frac{PS.k_{-3S}.k_{-4S}.(k_2+k_{-1})}{(k_{4S}+k_{-3S})} + D.S.k_1.\left(\frac{k_{3R}.(k_2+k_{4R})}{(k_{4R}+k_{-3R})}+\frac{k_{3S}.(k_2+k_{4S})}{(k_{4S}+k_{-3S})}\right) + D.Q.k_1.k_{-2} + \frac{PR.S.k_{-4R}.(k_2+k_{-1}).\left(k_{3R}.(k_{4S}+k_{-3S})+k_{3S}.(k_{4S}+k_{-3R})\right)}{(k_{4R}+k_{-3R}).(k_{4S}+k_{-3S})} + \frac{PS.S.k_{-4S}.(k_2+k_{-1}).\left(k_{3S}.(k_{4R}+k_{-3R})+k_{3R}.(k_{4R}+k_{-3S})\right)}{(k_{4R}+k_{-3R}).(k_{4S}+k_{-3S})} + \frac{PR.Q.k_{-2}.k_{-4R}.(k_{-1}+k_{-3R})}{(k_{4R}+k_{-3R})} + \frac{PS.Q.k_{-2}.k_{-4S}.(k_{-1}+k_{-3S})}{(k_{4S}+k_{-3S})}}$$

As before, exchanging every R for S and vice versa gives an expression for dPS/dt (the denominator is in fact again unchanged). Here the sums (k_{4R} + k_{-3R}) and (k_{4S} + k_{-3S}) appear frequently, often as denominators with products like $k_{3R} \cdot k_{4R}$ or $k_{-3R} \cdot k_{-4R}$ in the numerator. So there are similar pseudo-specificity constants like $k_{3R} \cdot k_{4R}/(k_{4R} + k_{-3R})$, this one for the reaction of the substituted enzyme E* with S to produce PR and the free enzyme E. Again as before, under initial rate conditions when PR, PS and Q are zero, the equation shows that the ratio of enantiomers formed is equal to the ratio of forward pseudo-specificity constants for R and S. Hence we define:

$$SCRf = \frac{k_{3R}.k_{4R}}{k_{4R}+k_{-3R}}$$

$$SCRb = \frac{k_{-3R}.k_{-4R}}{k_{4R}+k_{-3R}}$$

$$SCSf = \frac{k_{3S}.k_{4S}}{k_{4S}+k_{-3S}}$$

$$SCSb = \frac{k_{-3S}.k_{-4S}}{k_{4S}+k_{-3S}}$$

The backward constants SCRb and SCSb here are pseudo-specificity constants for the part reaction of the free enzyme E with PR or PS to produce S and substituted enzyme E*. The full rate expression also contains several appearances of the term (k_2 + k_{-1}), in a ratio with $k_1 \cdot k_2$. Hence it is appropriate to define also a pseudo-specificity constant for the achiral first part reaction of enzyme E with D to produce Q and substituted enzyme E*.

$$SCD = \frac{k_1.k_2}{k_2+k_{-1}}$$

By substituting all 5 of these pseudo-specificity constants we obtain the following rate expression:

$$\frac{dPR}{dt}=E_0.\frac{D.S.SCRf+\frac{S.PS.k_{3R}.SCSb}{SCD}-\frac{PR.Q.k_{-1}.k_{-2}.SCRb}{k_1.k_2}-\frac{PR.S.SCRb}{SCD}\left(SCSf+\frac{k_{3R}.SCSb}{k_{-4R}}\right)}{\begin{array}{c}D+S.\frac{SCRf+SCSf}{SCD}+\frac{Q.k_{-1}.k_{-2}}{k_1.k_2}+\frac{PR.SCRb}{SCD}+\frac{PS.SCSb}{SCD}\\+D.S.\left(\frac{SCRf.(k_2+k_{4R})}{k_2.k_{4R}}+\frac{SCSf.(k_2+k_{4S})}{k_2.k_{4S}}\right)+\frac{D.Q.k_{-2}}{k_2}\\+PR.S.\frac{SCRb}{SCD}.\left(\frac{SCSf}{k_{-3R}}+\frac{SCSf}{k_{4S}}+\frac{k_{3R}}{k_{-3R}}\right)+PS.S.\frac{SCSb}{SCD}.\left(\frac{SCRf}{k_{-3S}}+\frac{SCRf}{k_{4R}}+\frac{k_{3S}}{k_{-3S}}\right)\\+\frac{PR.Q.SCRb.k_{-2}.(k_{-1}+k_{-3R})}{k_1.k_2.k_{-3R}}+\frac{PS.Q.SCSb.k_{-2}.(k_{-1}+k_{-3S})}{k_1.k_2.k_{-3S}}\end{array}}$$

The rate constant k_{-4S} does not now appear separately. Other rate constants only appear in particular groups: k_1 as $k_1{\cdot}k_2/k_{-2}$; k_{-1} as $k_{-1}/(k_1{\cdot}k_2)$; k_{-2} as k_{-2}/k_2; k_{-4R} as k_{-4R}/k_{3R}.

Under initial rate conditions with Q, PR and PS all zero, this reduces to:

$$\frac{dPR}{dt}=\frac{E_0.D.S.SCRf}{D+S.\left(\frac{SCRf}{SCD}+\frac{SCSf}{SCD}\right)+D.S.\left(\frac{SCRf}{k_{4R}}+\frac{SCRf}{k_2}+\frac{SCSf}{k_{4S}}+\frac{SCSf}{k_2}\right)}$$

Comparison with the standard equation for ping-pong kinetics shows that the K_M values for substrates D and S are equal to:

$$K_{MS}=\frac{1}{\left(\frac{SCRf}{k_{4R}}+\frac{SCRf}{k_2}+\frac{SCSf}{k_{4S}}+\frac{SCSf}{k_2}\right)}$$

$$K_{MD}=\frac{\left(\frac{SCRf+SCSf}{SCD}\right)}{\left(\frac{SCRf}{k_{4R}}+\frac{SCRf}{k_2}+\frac{SCSf}{k_{4S}}+\frac{SCSf}{k_2}\right)}$$

The initial rate equation can also be used to calculate the ratio of E_0 to S_0 required to achieve a desired initial rate of increase in conversion, as used in comparing the progress for different parameter values.

Parameters that are defined as inputs to examine model behaviour include K_{eq}, enantiomer ratio E (= SCRf/SCSf), K_{MS}, and K_{MD}. Then k_2 is included as the rate constant fixed to set the timescale. Ratios of rate constants k_2/k_{4R} and $E{\cdot}k_{4S}/k_{4R}$ are used as parameters that are dimensionless and expected to be of the order of magnitude of 1, and have some support from the overall rate expression. They allow calculation of SCRf from K_{MS}, and then SCD using K_{MD} as well.

The parameter SCRf/b (= SCRf/SCRb) is dimensionless, and was found empirically to be very influential on the relative formation of the two enantiomers. It may tend to be larger for reactions with a larger overall K_{eq}, but the relationship will not be simple. For example, using an activated acyl donor in a transesterification reaction will raise K_{eq} via effects on k_1, k_2, k_{-1}, and k_{-2}. The second part reaction and hence SCRf/b will not be affected. We now use the relationship for equilibration of the two product enantiomers:

$$K_{rac}=1=\frac{k_{3R}.k_{4R}.k_{-3S}.k_{-4S}.}{k_{3S}.k_{4S}.k_{-3R}.k_{-4R}.}=\frac{SCRf.SCSb}{SCSf.SCRb}$$

which allows calculation of SCSb.

Now introduce the ratio k_{-1}/k_2, which accounts for all appearances of k_{-1} in the full rate expression, and allows calculation of k_{-1}. Then make use of the equilibrium constant K_{eq} for formation of PR and Q from D and S, which is related to rate constants by

$$K_{eq} = \frac{k_1.k_2.k_{3R}.k_{4R}}{k_{-1}.k_{-2}.k_{-3R}.k_{-4R}} = \frac{k_1.k_2.SCRf}{k_{-1}.k_{-2}.SCRb} = \frac{SCD.(k_2 + k_{-1}).SCRf}{k_{-1}.k_{-2}.SCRb}$$

which allows calculation of k_{-2}. Then using the definition of SCD we obtain k_1.

The final two input parameters are defined as the ratios k_{3R}/k_{-4R} and k_{3S}/k_{-4S}. These are dimensionless, and account for the only appearance of k_{-4R} in the overall rate expression (k_{-4S} does not appear at all). There may again be a tendency for these parameters to increase with K_{eq}, but this will certainly not be automatic, as explained for SCRf/b. Together with the known values of SCRf, SCRb, SCSf, and SCSb, these allow calculation of all remaining rate constants.

"Ping-pong, first" kinetics: diol ester hydrolysis

The most likely occurrence of this kinetic mechanism is for hydrolysis of the diesters of prochiral diols. In such cases, the water concentration in the reaction mixture will always be in large excess and hence essentially constant. Thus we use a pseudo-first order rate constant k_{3w} ($=k_3\cdot[H_2O]$) instead of the second order rate constant k_3. The pseudo-steady state approximation then leads to the following full rate expression:

$$\frac{1}{E_0}\frac{dPR}{dt} = \frac{\frac{S.k_{1R}.k_{2R}.k_{3w}.k_4}{(k_{2R}+k_{-1R})} + \frac{S.PS.k_{1R}.k_{2R}.k_{-1S}.k_{-2S}.(k_4+k_{-3})}{(k_{2R}+k_{-1R}).(k_{2S}+k_{-1S})} - \frac{PR.Q.k_{-1R}.k_{-2R}.k_{-3}.k_{-4}}{(k_{2R}+k_{-1R})} - \frac{PR.S.k_{1S}.k_{2S}.k_{-1R}.k_{-2R}.(k_4+k_{-3})}{(k_{2R}+k_{-1R}).(k_{2S}+k_{-1S})}}{\begin{array}{c} k_{3w}.k_4 + S.\left(\frac{k_{1R}.[k_{2R}.(k_4+k_{-3}) + k_{3w}.(k_{2R}+k_4)]}{(k_{2R}+k_{-1R})} + \frac{k_{1S}.[k_{2S}.(k_4+k_{-3}) + k_{3w}.(k_{2S}+k_4)]}{(k_{2S}+k_{-1S})}\right) \\ +Q.k_{-4}.(k_{3w}+k_{-3}) + \frac{PR.k_{-1R}.k_{-2R}.(k_4+k_{-3})}{k_{2R}+k_{-1R}} + \frac{PS.k_{-1S}.k_{-2S}.(k_4+k_{-3})}{k_{2S}+k_{-1S}} \\ +S.PR.k_{-2R}.(k_4+k_{-3}).\left(\frac{k_{1R}.(k_{2S}+k_{-1S}) + k_{1S}.(k_{2S}+k_{-1R})}{(k_{2R}+k_{-1R}).(k_{2S}+k_{-1S})}\right) \\ +S.PS.k_{-2S}.(k_4+k_{-3}).\left(\frac{k_{1S}.(k_{2R}+k_{-1R}) + k_{1R}.(k_{2R}+k_{-1S})}{(k_{2R}+k_{-1R}).(k_{2S}+k_{-1S})}\right) \\ + \frac{Q.PR.k_{-2R}.k_{-4}.(k_{-1R}+k_{-3})}{(k_{2R}+k_{-1R})} + \frac{Q.PS.k_{-2S}.k_{-4}.(k_{-1S}+k_{-3})}{(k_{2S}+k_{-1S})} \end{array}}$$

As before replacing every R with S and vice versa gives the expression for the rate of PS formation. Again as before the denominator is completely symmetrical and so unchanged on such a replacement. The frequent occurrence of sums ($k_{2R} + k_{-1R}$) and ($k_{2S} + k_{-1S}$), usually as divisors to products of rate constants, suggests again the definition of pseudo-specificity constants for the first half reaction of the enzyme with the diol diester S to produce monoester Q and the acyl enzyme intermediate E*.

$$SCRf = \frac{k_{1R}.k_{2R}}{k_{2R} + k_{-1R}}$$

$$SCRb = \frac{k_{-1R}.k_{-2R}}{k_{2R} + k_{-1R}}$$

$$SCSf = \frac{k_{1S}.k_{2S}}{k_{2S} + k_{-1S}}$$

$$SCSb = \frac{k_{-1S}.k_{-2S}}{k_{2S} + k_{-1S}}$$

Substituting these leads to the following expression:

$$\frac{dPR}{dt} = E_0.\frac{S.\frac{SCRf.k_{3w}.k_4}{k_4 + k_{-3}} + S.PS.SCRf.SCSb - PR.Q.\frac{SCRb.k_{-3}.k_{-4}}{k_4 + k_{-3}} - PR.S.SCRb.SCSf}{\begin{array}{c}\frac{k_{3w}.k_4}{k_4 + k_{-3}} + S.\left(SCRf.\left[1 + \frac{k_{3w}}{k_4 + k_{-3}}.\left(1 + \frac{k_4}{k_{2R}}\right)\right] + SCSf.\left[1 + \frac{k_{3w}}{k_4 + k_{-3}}.\left(1 + \frac{k_4}{k_{2S}}\right)\right]\right) \\ +Q.\frac{k_{-4}.(k_{3w} + k_{-3})}{k_4 + k_{-3}} + PR.SCRb + PS.SCSb + \\ +S.PR.\left(\frac{SCRb.SCSf}{k_{2S}} + \frac{SCRb.SCSf}{k_{-1R}} + \frac{k_{-2R}.SCRf}{k_{2R}}\right) \\ +S.PS.\left(\frac{SCRf.SCSb}{k_{2R}} + \frac{SCRf.SCSb}{k_{-1S}} + \frac{k_{-2S}.SCSf}{k_{2S}}\right) \\ +Q.PR.\frac{SCRb.k_{-4}}{(k_4 + k_{-3})}.\left(1 + \frac{k_{-3}}{k_{-1R}}\right) + Q.PS.\frac{SCSb.k_{-4}}{(k_4 + k_{-3})}.\left(1 + \frac{k_{-3}}{k_{-1S}}\right)\end{array}}$$

The rate constants k_{1R} and k_{1S} no longer appear individually in this expression.

Under initial rate conditions with Q, PR, and PS all zero, the expression reduces to:

$$\frac{dPR}{dt} = \frac{E_0.S.SCRf.k_{3w}.k_4}{k_{3w}.k_4 + S.\left(SCRf.\left[k_4 + k_{-3} + k_{3w}.\left(1 + \frac{k_4}{k_{2R}}\right)\right] + SCSf.\left[k_4 + k_{-3} + k_{3w}.\left(1 + \frac{k_4}{k_{2S}}\right)\right]\right)}$$

The denominator is unchanged on exchanging R and S, so the initial rates of formation of the two enantiomers are once again in the ratio SCRf/SCSf, i.e., E. From this initial rate expression, it is also clear that the K_M value (for S) is given by:

$$K_M = \frac{k_{3w}.k_4}{\left(SCRf.\left[k_4 + k_{-3} + k_{3w}.\left(1 + \frac{k_4}{k_{2R}}\right)\right] + SCSf.\left[k_4 + k_{-3} + k_{3w}.\left(1 + \frac{k_4}{k_{2S}}\right)\right]\right)}$$

As input parameters we again use the initial enantiomer ratio E (= SCRf/SCSf), the K_M value (for S), and the overall equilibrium constant K_{eq}. (Note that for this kinetic model, K_{eq} has dimensions, with units taken as M). Choose k_4 as the rate constant given a value to set the timescale.

To proceed, define as input parameters two ratios of rate constants, k_{2R}/k_4 and $k_{2R}/(E \cdot k_{2S})$. Both these are dimensionless, and are expected to be of the order of magnitude of 1. The ratio k_{2R}/k_4 also appears in the overall rate expression. Define also k_{3w}/k_4, which is dimensionless, likely to be of the order of magnitude of 1, and has some support from the overall rate expression.

The ratio k_{-4}/k_4 also has some support from the overall rate expression, but has dimensions of M^{-1}. Hence we actually define the input parameter as $k_{-4} \cdot K_{eq}/k_4$, which is dimensionless. It will often be of the order of 1, but could deviate significantly depending on whether the first or second stage of the reaction dominates the overall equilibrium. The ratio SCRf/b (=SCRf/SCRb) is again found empirically to be very important in determining the behaviour. Then make use of the following relationship of the overall equilibrium constant

$$K_{eq} = \frac{k_{1R}.k_{2R}.k_{3w}.k_4}{k_{-1R}.k_{-2R}.k_{-3}.k_{-4}} = \frac{SCRf.k_{3w}.k_4}{SCRb.k_{-3}.k_{-4}}$$

Now using K_{eq}, k_4, k_{3w}/k_4, $k_{-4}\cdot K_{eq}/k_4$ and SCRf/b, we can calculate k_{3w}, k_{-4} and then k_{-3}. Then using k_{2R}/k_4, $k_{2R}/(E\cdot k_{2S})$, E and K_M, we can calculate k_{2R}, k_{2S} and then SCRf and SCSf (the latter using the expression above for K_M).

For equilibration of the product enantiomers we have

$$K_{rac} = 1 = \frac{k_{1R}.k_{2R}.k_{-1S}.k_{-2S}.}{k_{1S}.k_{2S}.k_{-1R}.k_{-2R}.} = \frac{SCRf.SCSb}{SCSf.SCRb}$$

Hence using SCRf/b again we obtain SCSb.

Finally, define two more ratios of rate constants, k_{-1R}/k_{-3} and $E\cdot k_{-1S}/k_{-3}$, which appear in the overall rate expression. These are dimensionless, and may be of the order of 1, but a very different value is also possible. With the values of 4 SC's and 2 rate constants for the first part reaction, these two final parameters allow calculation of all remaining rate constants.

"Ping-pong, both" kinetics: diacid case

For this kinetic model there are no less than 16 separate elementary rate constants, and 8 different enzyme forms. Using the quasi-steady state approximation it is possible to derive the following expression for the rate of product formation:

$$
\frac{1}{E_0}\frac{dPR}{dt}=\frac{\begin{aligned}&\frac{S.B^2.k_{1R}.k_{2R}.k_{3R}.k_{4R}.k_{3S}.k_{4S}}{(k_{2R}+k_{-1R}).(k_{4R}+k_{-3R}).(k_{4S}+k_{-3S})}+\frac{S.B.Q.k_{1R}.k_{2R}.k_{3R}.k_{4R}.k_{-1S}.k_{-2S}}{(k_{2R}+k_{-1R}).(k_{2S}+k_{-1S}).(k_{4R}+k_{-3R})}\\&-\frac{PR.B.Q.k_{3S}.k_{4S}.k_{-1R}.k_{-2R}.k_{-3R}.k_{-4R}}{(k_{2R}+k_{-1R}).(k_{4R}+k_{-3R}).(k_{4S}+k_{-3S})}-\frac{PR.Q^2.k_{-1R}.k_{-2R}.k_{-3R}.k_{-4R}.k_{-1S}.k_{-2S}}{(k_{2R}+k_{-1R}).(k_{2S}+k_{-1S}).(k_{4R}+k_{-3R})}\end{aligned}}{\begin{aligned}&S.B.\left(\frac{k_{1R}.k_{2R}.k_{3S}.k_{4S}}{(k_{2R}+k_{-1R}).(k_{4S}+k_{-3S})}+\frac{k_{1S}.k_{2S}.k_{3R}.k_{4R}}{(k_{2S}+k_{-1S}).(k_{4R}+k_{-3R})}\right)\\&+\frac{S.B^2.k_{3R}.k_{3S}}{(k_{4R}+k_{-3R}).(k_{4S}+k_{-3S})}.\left(\frac{k_{1R}.k_{4S}.(k_{2R}+k_{4R})}{(k_{2R}+k_{-1R})}+\frac{k_{1S}.k_{4R}.(k_{2S}+k_{4S})}{(k_{2S}+k_{-1S})}\right)\\&+S.Q.\frac{k_{1R}.k_{2R}.k_{-1S}.k_{-2S}+k_{1S}.k_{2S}.k_{-1R}.k_{-2R}}{(k_{2R}+k_{-1R}).(k_{2S}+k_{-1S})}+S.Q^2.\frac{k_{-2R}.k_{-2S}.(k_{1R}.k_{-1S}+k_{1S}.k_{-1R})}{(k_{2R}+k_{-1R}).(k_{2S}+k_{-1S})}\\&+S.B.Q.\left(\begin{aligned}&\frac{k_{3R}.k_{-2S}.[k_{1R}.k_{-1S}.(k_{2R}+k_{4R})+k_{1S}.k_{4R}.(k_{2R}+k_{-1R})]}{(k_{2R}+k_{-1R}).(k_{2S}+k_{-1S}).(k_{4R}+k_{-3R})}\\&+\frac{k_{3S}.k_{-2R}.[k_{1S}.k_{-1R}.(k_{2S}+k_{4S})+k_{1R}.k_{4S}.(k_{2S}+k_{-1S})]}{(k_{2R}+k_{-1R}).(k_{2S}+k_{-1S}).(k_{4S}+k_{-3S})}\end{aligned}\right)\\&\frac{B^2.k_{3R}.k_{4R}.k_{3S}.k_{4S}}{(k_{4R}+k_{-3R}).(k_{4S}+k_{-3S})}+\frac{Q^2.k_{-1R}.k_{-2R}.k_{-1S}.k_{-2S}}{(k_{2R}+k_{-1R}).(k_{2S}+k_{-1S})}\\&+B.Q.\left(\frac{k_{3R}.k_{4R}.k_{-1S}.k_{-2S}}{(k_{2S}+k_{-1S}).(k_{4R}+k_{-3R})}+\frac{k_{3S}.k_{4S}.k_{-1R}.k_{-2R}}{(k_{2R}+k_{-1R}).(k_{4S}+k_{-3S})}\right)\\&+\frac{PR.B.k_{3S}.k_{4S}.k_{-3R}.k_{-4R}}{(k_{4R}+k_{-3R}).(k_{4S}+k_{-3S})}+\frac{PS.B.k_{3R}.k_{4R}.k_{-3S}.k_{-4S}}{(k_{4R}+k_{-3R}).(k_{4S}+k_{-3S})}\\&+\frac{PR.B^2.k_{3R}.k_{3S}.k_{4S}.k_{-4R}}{(k_{4R}+k_{-3R}).(k_{4S}+k_{-3S})}+\frac{PS.B^2.k_{3S}.k_{3R}.k_{4R}.k_{-4S}}{(k_{4R}+k_{-3R}).(k_{4S}+k_{-3S})}\\&+\frac{PR.Q.k_{-3R}.k_{-4R}.k_{-1S}.k_{-2S}}{(k_{2S}+k_{-1S}).(k_{4R}+k_{-3R})}+\frac{PS.Q.k_{-3S}.k_{-4S}.k_{-1R}.k_{-2R}}{(k_{2R}+k_{-1R}).(k_{4S}+k_{-3S})}\\&+\frac{PR.Q^2.k_{-1S}.k_{-2S}.k_{-2R}.k_{-4R}.(k_{-1R}+k_{-3R})}{(k_{2R}+k_{-1R}).(k_{2S}+k_{-1S}).(k_{4R}+k_{-3R})}+\frac{PS.Q^2.k_{-1R}.k_{-2R}.k_{-2S}.k_{-4S}.(k_{-1S}+k_{-3S})}{(k_{2R}+k_{-1R}).(k_{2S}+k_{-1S}).(k_{4S}+k_{-3S})}\\&+\frac{PR.B.Q.k_{-4R}}{(k_{4R}+k_{-3R})}.\left(\frac{k_{3R}.k_{-1S}.k_{-2S}}{(k_{2S}+k_{-1S})}+\frac{k_{3S}.k_{4S}.k_{-2R}.(k_{-1R}+k_{-3R})}{(k_{2R}+k_{-1R}).(k_{4S}+k_{-3S})}\right)\\&+\frac{PS.B.Q.k_{-4S}}{(k_{4S}+k_{-3S})}.\left(\frac{k_{3S}.k_{-1R}.k_{-2R}}{(k_{2R}+k_{-1R})}+\frac{k_{3R}.k_{4R}.k_{-2S}.(k_{-1S}+k_{-3S})}{(k_{2S}+k_{-1S}).(k_{4R}+k_{-3R})}\right)\end{aligned}}
$$

As with the diol ester hydrolysis case, this expression contains multiple occurrences of the groups ($k_{2R}+k_{-1R}$) and ($k_{2S}+k_{-1S}$). (Their total number could be reduced slightly by cancellation top and bottom, but they are retained as shown here because of the next step.) They appear with products of rate constants that suggest the definition of pseudo-specificity constants as before:

$$SCRf=\frac{k_{1R}.k_{2R}}{k_{2R}+k_{-1R}}$$

$$SCRb=\frac{k_{-1R}.k_{-2R}}{k_{2R}+k_{-1R}}$$

$$SCSf=\frac{k_{1S}.k_{2S}}{k_{2S}+k_{-1S}}$$

$$SCSb=\frac{k_{-1S}.k_{-2S}}{k_{2S}+k_{-1S}}$$

But in this case there are also frequent occurrences of ($k_{4R}+k_{-3R}$) and ($k_{4S}+k_{-3S}$). These naturally suggest a further set of pseudo-specificity constants, which apply to the second stage part reaction converting the substituted enzyme E* (acyl enzyme intermediate) back to the free enzyme, liberating either the free acid or the ester. These are defined as follows:

$$SCR2f = \frac{k_{3R}.k_{4R}}{k_{4R} + k_{-3R}}$$

$$SCR2b = \frac{k_{-3R}.k_{-4R}}{k_{4R} + k_{-3R}}$$

$$SCS2f = \frac{k_{3S}.k_{4S}}{k_{4S} + k_{-3S}}$$

$$SCS2b = \frac{k_{-3S}.k_{-4S}}{k_{4S} + k_{-3S}}$$

Substituting all these SC's leads to the expression:

$$\frac{1}{E_0}\frac{dPR}{dt} = $$

$$= \frac{\begin{matrix} S.B^2.SCRf.SCR2f.SCS2f + S.B.Q.SCRf.SCR2f.SCSb \\ -PR.B.Q.SCS2f.SCRb.SCR2b - PR.Q^2.SCRb.SCR2b.SCSb \end{matrix}}{\begin{matrix} S.B.(SCRf.SCS2f + SCSf.SCR2f) + S.B^2.SCR2f.SCS2f.\left(SCRf.\left(\frac{1}{k_{2R}} + \frac{1}{k_{4R}}\right) + SCSf.\left(\frac{1}{k_{2S}} + \frac{1}{k_{4S}}\right)\right) \\ +S.Q.(SCRf.SCSb + SCSf.SCRb) + S.Q^2.SCRb.SCSb.\left(\frac{k_{1R}}{k_{-1R}} + \frac{k_{1S}}{k_{-1S}}\right) \\ +S.B.Q.\left(SCR2f.SCSb.\left(SCRf.\left(\frac{1}{k_{2R}} + \frac{1}{k_{4R}}\right) + \frac{k_{1S}}{k_{-1S}}\right) + SCS2f.SCRb.\left(SCSf.\left(\frac{1}{k_{2S}} + \frac{1}{k_{4S}}\right) + \frac{k_{1R}}{k_{-1R}}\right)\right) \\ +B^2.SCR2f.SCS2f + Q^2.SCRb.SCSb + B.Q.(SCR2f.SCSb + SCS2f.SCRb) + PR.B.SCS2f.SCR2b \\ +PS.B.SCR2f.SCS2b + \frac{PR.B^2.SCR2f.SCS2f.k_{-4R}}{k_{4R}} + \frac{PS.B^2.SCR2f.SCS2f.k_{-4S}}{k_{4S}} \\ +PR.Q.SCR2b.SCSb + PS.Q.SCS2b.SCRb + PR.Q^2.SCSb.SCRb.SCR2b.\left(\frac{1}{k_{-1R}} + \frac{1}{k_{-3R}}\right) \\ +PS.Q^2.SCSb.SCRb.SCS2b.\left(\frac{1}{k_{-1S}} + \frac{1}{k_{-3S}}\right) \\ +PR.B.Q.\left(\frac{SCSb.SCR2f.k_{-4R}}{k_{4R}} + SCRb.SCR2b.SCS2f.\left(\frac{1}{k_{-1R}} + \frac{1}{k_{-3R}}\right)\right) \\ +PS.B.Q.\left(\frac{SCRb.SCS2f.k_{-4S}}{k_{4S}} + SCSb.SCS2b.SCR2f.\left(\frac{1}{k_{-1S}} + \frac{1}{k_{-3S}}\right)\right) \end{matrix}}$$

Several rate constants no longer appear individually in this expression: k_{3R}, k_{3S}, k_{-1R}, k_{-1S}. Others appear only in fixed combinations: k_{1R} and k_{1S} only in the ratios k_{1R}/k_{-1R} and k_{1S}/k_{-1S}; k_{-4R} and k_{-4S} only in the ratios k_{-4R}/k_{4R} and k_{-4S}/k_{4S}; k_{-3R} and k_{-3S} only in the groupings ($1/k_{-1R} + 1/k_{-3R}$) and ($1/k_{-1S} + 1/k_{-3S}$); k_{2R} and k_{2S} only in the groupings ($1/k_{2R} + 1/k_{4R}$) and ($1/k_{2S} + 1/k_{4S}$).

For this kinetic model, the parameter values that might be known differ depending on the type of reaction involved. The most common would be either hydrolysis or synthesis of prochiral diacid esters (in the latter case from the free acid by direct reversal of hydrolysis).

In the case of hydrolysis, an initial rate can be estimated under conditions where Q, PR and PS are all equal to zero. B, which here is H_2O, can be taken as being in constant large excess. This leads to an initial rate of

$$\frac{dPR}{dt} = \frac{E_0.S.SCRf}{1 + S.\left(SCRf.\left(\frac{1}{k_{2R}} + \frac{1}{k_{4R}}\right) + SCSf.\left(\frac{1}{k_{2S}} + \frac{1}{k_{4S}}\right) + \frac{SCRf.}{B.SCR2f} + \frac{SCSf}{B.SCS2f}\right)}$$

Once again the denominator is unchanged on exchange of R and S, so the ratio of initial rates of formation of the enantiomers is SCRf/SCSf, defined again as E. This expression also shows that the apparent K_M value for S would be found as:

$$K_M = \frac{1}{SCRf.\left(\frac{1}{k_{2R}} + \frac{1}{k_{4R}}\right) + SCSf.\left(\frac{1}{k_{2S}} + \frac{1}{k_{4S}}\right) + \frac{SCRf.}{B.SCR2f} + \frac{SCSf}{B.SCS2f}}$$

This will be an apparent K_M value, dependent on the concentration of B (H_2O), although this will normally be in constant large excess.

But in the case of ester synthesis, even under initial rate conditions, Q (H_2O) will not generally be zero, just PR and PS. Hence:

$$\frac{dPR}{dt} = \frac{E_0.S.SCRf.\left(B + \frac{Q.SCSb}{SCS2f}\right)}{\begin{array}{c} S.\left(\frac{SCRf}{SCR2f} + \frac{SCSf}{SCS2f}\right) + S.B.\left(SCRf.\left(\frac{1}{k_{2R}} + \frac{1}{k_{4R}}\right) + SCSf.\left(\frac{1}{k_{2S}} + \frac{1}{k_{4S}}\right)\right) \\ + \frac{S.Q}{B}.\left(\frac{SCRf.SCSb + SCSf.SCRb}{SCR2f.SCS2f}\right) + \frac{S.Q^2}{B}.\frac{SCRb.SCSb}{SCR2f.SCS2f}.\left(\frac{k_{1R}}{k_{-1R}} + \frac{k_{1S}}{k_{-1S}}\right) \\ +S.Q.\left(\frac{SCSb}{SCS2f}.\left[SCRf.\left(\frac{1}{k_{2R}} + \frac{1}{k_{4R}}\right) + \frac{k_{1S}}{k_{-1S}}\right] + \frac{SCRb}{SCR2f}.\left[SCSf.\left(\frac{1}{k_{2S}} + \frac{1}{k_{4S}}\right) + \frac{k_{1R}}{k_{-1R}}\right]\right) \\ +B + \frac{Q^2}{B}.\frac{SCRb.SCSb}{SCR2f.SCS2f} + Q.\left(\frac{SCSb}{SCS2f} + \frac{SCRb}{SCR2f}\right) \end{array}}$$

This expression indicates that the effect of concentration B on the rate will not follow a simple hyperbolic (Michaelis–Menten) relationship. At fixed concentration of B and Q, it would be possible to estimate an apparent K_M for S, but this would be a function of both these concentrations (of B and Q). So it was judged that any information on apparent K_M would probably not be useful in analysing the behaviour.

Again the denominator stays exactly the same on exchanging R and S, so we derive for the ratio of initial rates

$$\frac{Initial\ rate\ R}{Initial\ rate\ S} = \frac{SCRf.\left(B + \frac{Q.SCSb}{SCS2f}\right)}{SCSf.\left(B + \frac{Q.SCRb}{SCR2f}\right)} = \frac{SCRf.\left(\frac{B}{Q} + \frac{SCSb}{SCS2f}\right)}{SCSf.\left(\frac{B}{Q} + \frac{SCRb}{SCR2f}\right)}$$

If B/Q is large enough, then this ratio of rates is close to SCRf/SCSf (i.e. E) as in the previous cases. However, the molar concentration of Q (H_2O) will often be considerably more than that of B (alcohol). The ratios SCSb/SCS2f and SCRb/SCR2f will depend on enzyme characteristics, and may be small, making the limiting case more likely. But if they are significant compared with B/Q, then the initial ratio of enantiomer formation may be noticeably different from E. Interestingly, depending on the enzyme characteristics, the ratio of enantiomers might be higher or lower than E.

As a result of these differences, the choices of input parameters are slightly different for the hydrolysis and esterification cases. For hydrolysis, we again use the initial enantiomer ratio E (= SCRf/SCSf), the apparent K_M value (for S, at fixed B concentration), and the overall equilibrium constant K_{eq}. (For consistency with the esterification case, K_{eq} is here taken as the dimensionless value with its definition including the concentration of H_2O, so its value might be quite small.) Then choose k_{2R} as the rate constant assigned a value to set the timescale.

Now define a first set of input parameters based on ratios of rate constants: k_{4R}/k_{2R}, $k_{2R}/(E\cdot k_{2S})$ and k_{4S}/k_{2S}. Each of these is dimensionless, and expected to be of the order of magnitude of 1. The overall rate expression also includes groups where k_{2R} and k_{4R}, and k_{2S} and k_{4S}, appear together.

To make use of the expression for the apparent K_M, we need relationships for SCR2f and SCS2f. Define E2 (= SCR2f/SCS2f), but actually use E2/E as the dimensionless input parameter. It will often be of the order of 1, but possibly far away if the enantiopreference of the enzyme is very different in the acylation and de-acylation phases of the reaction. Also define as a parameter the dimensionless ratio SCR2f/SCRf. This could also be of the order of 1, but could be considerably smaller and still give a sensible reaction progress, because B (H_2O) >> S. With these two parameters set, SCRf can be calculated from the K_M value.

Now introduce SCRf/b (=SCRf/SCRb), which again will be found empirically to be very important in determining the behaviour. Also define the parameter SCSf/b (=SCSf/SCSb). Both these parameters will have some tendency to increase with K_{eq}, but the relationship will not be simple, because the favourability may differ substantially for the two part reactions of forming and then breaking down the E* intermediate.

Now use the two equilibrium relationships to find all remaining SC's. Racemisation here requires all 4 reaction steps for each enantiomer:

$$K_{rac} = 1 = \frac{k_{1R}.k_{2R}.k_{3R}.k_{4R}.k_{-1S}.k_{-2S}.k_{-3S}.k_{-4S}}{k_{1S}.k_{2S}.k_{3S}.k_{4S}.k_{-1R}.k_{-2R}.k_{-3R}.k_{-4R}} = \frac{SCRf.SCR2f.SCSb.SCS2b}{SCSf.SCS2f.SCRb.SCR2b}$$

While the overall reaction equilibrium constant

$$K_{eq} = \frac{k_{1R}.k_{2R}.k_{3R}.k_{4R}}{k_{-1R}.k_{-2R}.k_{-3R}.k_{-4R}} = \frac{SCRf.SCR2f}{SCRb.SCR2b}$$

We now need 4 more parameters, one for each part reaction and enantiomer. From the overall rate expression, the ratios k_{1R}/k_{-1R}, k_{1S}/k_{-1S}, k_{4R}/k_{-4R}, k_{4S}/k_{-4S} look attractive, but they all have dimensions because one of the rate constants is second order. So it is preferred to use dimensionless ratios like k_{-1R}/k_{2R}, which can be seen as controlling the partitioning of the intermediate ESR. Hence we use the following ratios as input parameters: k_{-1R}/k_{2R}, k_{-1S}/k_{2S}, k_{-3R}/k_{4R} and k_{-3S}/k_{4S}. For each part reaction and enantiomer, we now have 1 rate constant, two SC's and this final parameter, allowing all rate constants to be calculated.

In the case of an esterification reaction, we again use the overall equilibrium constant K_{eq} as one input parameter, along with k_{2R} as the rate constant that sets the timescale. The ratio E (= SCRf/SCSf) is also retained, even though in this case it may not give the initial ratio of enantiomer formation. The true dissociation constant for the $E\cdot S_R$ complex ($K_S = k_{-1R}/k_{1R}$) was chosen as in input instead of any apparent K_M value. It is unlikely to be known accurately, but it will often be possible to make a reasonable order of magnitude estimate, perhaps making use of kinetic parameters. This ratio of rate constants also appears in the full rate expression.

Now select the parameter k_{-1R}/k_{2R} as in the hydrolysis case, and SCRf/b (=SCRf/SCRb), again found empirically to be an influential parameter. Combining with k_{2R} and K_S then allows calculation of k_{1R}, k_{-1R}, k_{-2R} and both SCRf and SCRb. Then using E we get SCSf.

To proceed, define 3 more parameters as used in the hydrolysis case and discussed there: E2/E, SCR2f/SCRf, and SCSf/b (=SCSf/SCSb). With values of SCRf, SCRb and SCSf, together with the relationships to K_{rac} and K_{eq} as in the hydrolysis case, we can now calculate all 8 SC's.

For the first part reaction with the S enantiomer, we have SCSf and SCSb. Using two more parameters, k_{2R}/(E·k_{2S}) and k_{-1S}/k_{2S}, as for the hydrolysis case (again discussed there), allows calculation of all 4 rate constants. For the second part reaction with the R enantiomer, choose another two parameters as used and discussed for hydrolysis: k_{4R}/k_{2R} and k_{-3R}/k_{4R}. Combined with SCR2f and SCR2b, all rate constants are determined. For the S enantiomer, we use k_{-3S}/k_{4S} as for hydrolysis, but it seems more consistent to select k_{4R}/(E·k_{4S}) as the final parameter (not k_{4S}/k_{2S}).

Estimation of ee at 95% of equilibrium conversion

This was made by interpolation of the table of conversion and product ee output from the calculated progress simulation. The K_{eq} value is defined for the reaction to produce the R enantiomer (arbitrarily taken as being the predominant one). Hence the "equilibrium" conversion is calculated as that which would be achieved if the enzyme were completely enantiospecific. To the extent that S product is also formed, the observed conversion can exceed this "equilibrium" value. (At final true equilibrium there would be equal concentrations of the two enantiomers, but of course this equilibrium should not be approached in a preparative reaction.) Formation of the unfavoured enantiomer will make reaching 95% of equilibrium conversion rather easier – if formation of 95% of the equilibrium concentration of the favoured enantiomer were required, the ee value found would be even lower.
